# Higher host plant specialization of root‐associated endophytes than mycorrhizal fungi along an arctic elevational gradient

**DOI:** 10.1002/ece3.6604

**Published:** 2020-08-06

**Authors:** Nerea Abrego, Tea Huotari, Ayco J. M. Tack, Björn D. Lindahl, Gleb Tikhonov, Panu Somervuo, Niels Martin Schmidt, Otso Ovaskainen, Tomas Roslin

**Affiliations:** ^1^ Department of Agricultural Sciences University of Helsinki Helsinki Finland; ^2^ Centre for Biodiversity Dynamics Department of Biology Norwegian University of Science and Technology Trondheim Norway; ^3^ Department of Ecology Environment and Plant Sciences Stockholm University Stockholm Sweden; ^4^ Department of Soil and Environment Swedish University of Agricultural Sciences Uppsala Sweden; ^5^ Organismal and Evolutionary Biology Research Programme University of Helsinki Helsinki Finland; ^6^ Computational Systems Biology group Department of Computer Science Aalto University Espoo Finland; ^7^ Arctic Research Centre Department of Bioscience Aarhus University Roskilde Denmark; ^8^ Department of Ecology Swedish University of Agricultural Sciences Uppsala Sweden

**Keywords:** Arctic, elevation gradient, endophytic fungi, joint species distribution model, mycorrhizal network, specialization

## Abstract

How community‐level specialization differs among groups of organisms, and changes along environmental gradients, is fundamental to understanding the mechanisms influencing ecological communities. In this paper, we investigate the specialization of root‐associated fungi for plant species, asking whether the level of specialization varies with elevation. For this, we applied DNA barcoding based on the ITS region to root samples of five plant species equivalently sampled along an elevational gradient at a high arctic site. To assess whether the level of specialization changed with elevation and whether the observed patterns varied between mycorrhizal and endophytic fungi, we applied a joint species distribution modeling approach. Our results show that host plant specialization is not environmentally constrained in arctic root‐associated fungal communities, since there was no evidence for changing specialization with elevation, even if the composition of root‐associated fungal communities changed substantially. However, the level of specialization for particular plant species differed among fungal groups, root‐associated endophytic fungal communities being highly specialized on particular host species, and mycorrhizal fungi showing almost no signs of specialization. Our results suggest that plant identity affects associated mycorrhizal and endophytic fungi differently, highlighting the need of considering both endophytic and mycorrhizal fungi when studying specialization in root‐associated fungal communities.

## Graphical abstract

We investigated the specialization of root‐associated fungi (RAF) for plant species, asking whether the level of specialization varies with elevation. For this, we acquired molecular data on root‐associated fungi of five plant species equivalently sampled along an elevational gradient at a high arctic site. Our results show that host plant specialization is not environmentally constrained in root‐associated fungal communities, since there was no evidence for changing specialization along elevation, even if the composition of root‐associated fungal communities changed substantially.

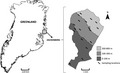

## 1 INTRODUCTION

Measuring specialization in interaction networks has important implications for understanding co‐evolutionary dynamics (Thrall, Hochberg, Burdon, & Bever, 2007) and the mechanisms shaping species distributions (Mariadassou, Pichon, & Ebert, 2015). Network‐level specialization of interaction networks is often compared along geographic and environmental gradients to understand macroecological and biogeographical patterns of biodiversity (Devoto, Medan, & Montaldo, 2005; Olesen & Jordano, 2002; Schleuning et al., 2012). However, most of these large‐scale studies assume specialization to be a constant network‐level trait, even if specialization in interaction networks can depend on the local environmental conditions (Cobian, Egan, & Amend, 2019; Pellissier et al., 2018). Certain environmental conditions can exert higher selection pressure on interaction partners with specific ecological functions, resulting in species interacting with a more specific subset of partner species. In particular, there is growing evidence that specialization is a plastic trait in the case of the microbial communities associated with plants (Cobian et al., 2019).

Root‐associated fungi (RAF) comprise highly species rich communities that have important consequences on the fitness of the associated plants (Bacon & White, 2000; Smith & Read, 2008). These associations are of particular importance in arctic environments with low nutrient availability (Hobbie & Hobbie, 2006). Interestingly, arctic RAF communities tend to show low levels of specialization as compared to tropical, temperate, and boreal ecosystems (Botnen et al., 2014; Ryberg, Andreasen, & Björk, 2011; Ryberg, Larsson, & Molau, 2009; Timling et al., 2012). Some authors (Botnen et al., 2014; Ryberg et al., 2009) have attributed these patterns to the scarce resources of arctic ecosystems, suggesting that by associating with generalist fungi rather than with specialist fungi, plants may colonize a wider range of habitats.

Many RAF are specialized on particular host plant(s) (e.g., Jacquemyn et al., 2014; Rochet, Moreau, Manzi, & Gardes, 2011), and thus vegetation or ecosystem type is generally an important determinant of the RAF community composition (Martínez‐García, Richardson, Tylianakis, Peltzer, & Dickie, 2015; Tedersoo et al., 2014). However, the level of specialization of RAF on the host plant differs greatly among fungal species (Tedersoo, Sadam, Zambrano, Valencia, & Bahram, 2010; Toju, Yamamoto, Sato, & Tanabe, 2013; Vandenkoornhuyse, Ridgway, Watson, Fitter, & Young, 2003) and, although there is not a consensus, also among functional fungal groups. While mycorrhizal fungi have generally been found to be highly determined by the host plant species (Sepp et al., 2019; Tedersoo et al., 2008; Tedersoo et al., 2010; Vandenkoornhuyse et al., 2003), root‐associated endophytic fungi have been found to have broad host ranges (Knapp, Pintye, & Kovács, 2012; Mandyam, Fox, & Jumpponen, 2012). Contrastingly, in a recent reassessment based on 111 previously published datasets, Põlme et al. (2018) did not find significant differences in the host specialization patterns of endophytic fungi compared to mycorrhizal fungi. The differences in the results among studies most likely arise from the lack of standardized sampling schemes in which the same plant species are equivalently sampled among locations.

The differences in the specialization patterns between mycorrhizal and endophytic fungi may lie on the manner in which they interact with the plant host. Mycorrhizal fungi develop specific adaptive structures to colonize plant roots (Smith & Read, 2008), whereas endophytic fungi do not develop joint interaction structures with the plant roots and can colonize other parts of the plant (Peterson, Wagg, & Pautler, 2008, but see Lukešová, Kohout, Větrovský, & Vohník, 2015). However, endophytic communities inhabiting different plant tissues show high levels of specificity and thus there is little evidence of fungal transmission through plant tissues (Cregger et al., 2018; Wearn, Sutton, Morley, & Gange, 2012).

Elevational gradients offer convenient systems for evaluating the effects of temperature and resource availability on biotic interaction structure at local scales (Körner, 2007). Changes in RAF community composition along elevational gradients have been variously attributed to changes in the distributions of the plants with which they are associated, and to abiotic stress such as thinning of soil depth, limited nutrient availability, and colder climatic conditions at higher elevations which restrict the occurrences of some species (Bahram, Põlme, Kõljalg, Zarre, & Tedersoo, 2012; Jarvis, Woodward, & Taylor, 2015; Kivlin, Lynn, Kazenel, Beals, & Rudgers, 2017; Matsuoka, Mori, Kawaguchi, Hobara, & Osono, 2016; Miyamoto, Nakano, Hattori, & Nara, 2014). Therefore, altitudinal gradients offer a convenient system for testing whether the specialization patterns detected across latitudes also apply to smaller scales within latitudes, that is, whether the level of specialization of RAF on plant species decreases with decreasing nutrient and water availability along local environmental gradients.

The overall aim of the present study was to investigate the host plant specialization patterns in RAF communities in an arctic setting. More specifically, we ask (a) whether host plant specialization differs between mycorrhizal and root‐associated endophytic communities and (b) whether the level of specialization of RAF on plant species changes along elevation. For this purpose, we acquired molecular data on RAF of five plant species that were equivalently sampled along an elevational gradient in the Zackenberg Valley (Northeast Greenland). We used joint species distribution models to quantitatively assess the amount of variation that the host plant identity explained over the RAF networks, as well as to determine whether the community‐level specialization of RAF on specific plant species depended on elevation. Based on earlier studies, we hypothesized that mycorrhizal fungi would be more specialized with respect to host plant species than root‐associated endophytic fungi. We also hypothesized a lower specialization of plant–RAF associations at higher elevations, because a lower degree of specialization could improve colonization in harsh climatic conditions and with low nutrient availability, in which the impact of RAF associations on plant survival can be expected to be especially critical.

## 2 MATERIALS AND METHODS

### 2.1 Study area and study design

**FIGURE 1:**
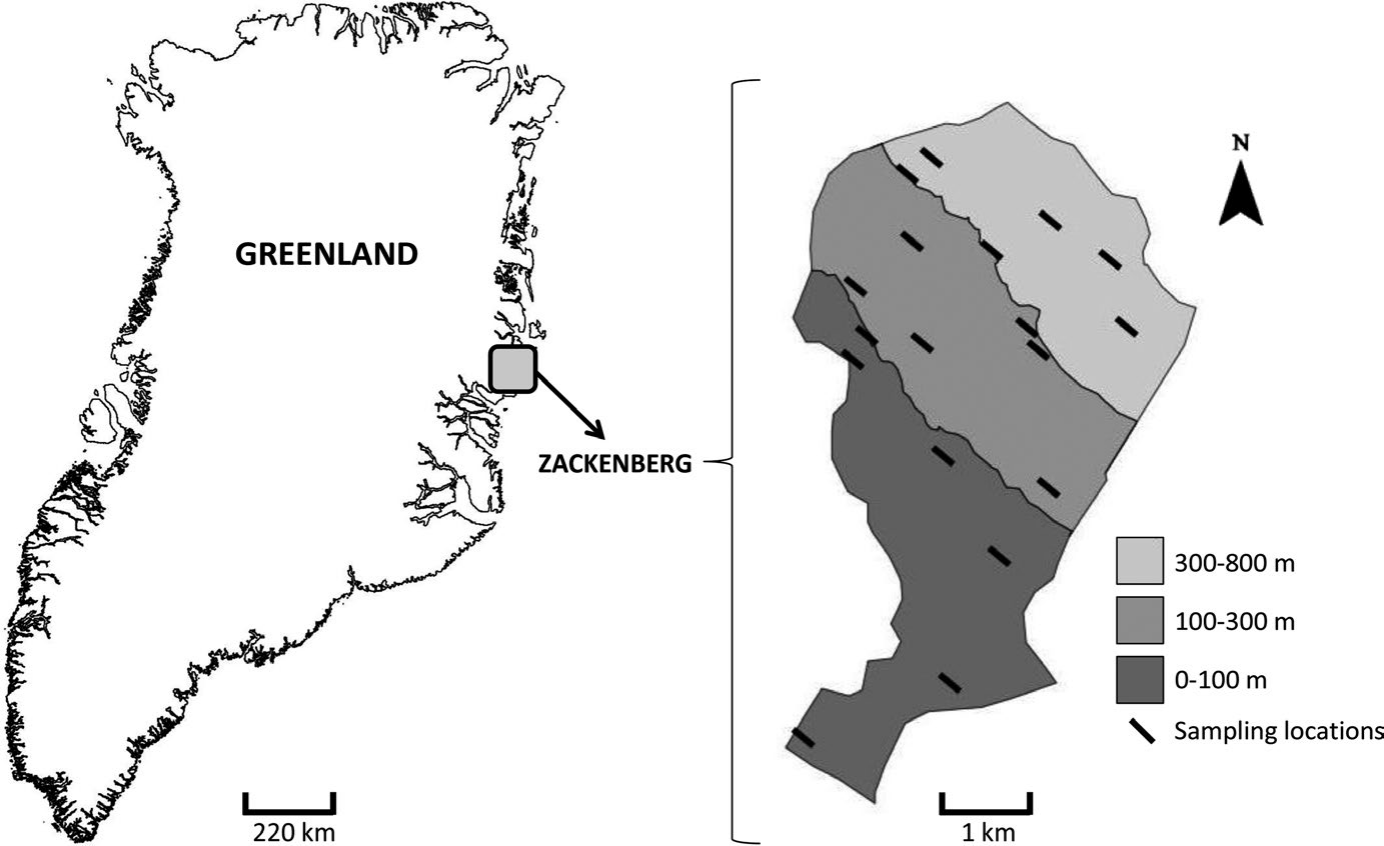
Location of Zackenberg Valley in Greenland (left) and spatial arrangement of the sampling locations on the eastern hillside of the Zackenberg Valley (right). The shaded areas correspond to the three elevational zones used in the sampling design for ensuring the uniform distribution of the sampling locations across the elevational gradient

The study area is located on the eastern hillside of the Zackenberg Valley, situated in Northeast Greenland (74°30′N/21°00′W, Figure 1a). The area belongs to the high arctic climate zone, characterized by mean monthly temperatures ranging from −20 to +7°C and an annual precipitation of 260 mm. The vegetation of the Zackenberg Valley consists of low tundra species, of which the arctic willow (*Salix arctica*), the arctic bell‐heather (*Cassiope tetragona),* and the mountain avens (*Dryas*) are some of the most abundant.

We selected five focal plant species, which were known to be relatively common along the whole elevational gradient (Bay, 1998): (a) Alpine bistort (*Bistorta vivipara*), (b) Mountain avens (*Dryas*), (c) Arctic willow (*Salix arctica*), (d) Purple saxifrage (*Saxifraga oppositifolia*), and (e) Moss campion (*Silene acaulis*). We note that the two dominant species of *Dryas* (*Dryas octopetala* and *D. integrifolia*) crossbreed and that most of those in northeastern Greenland are hybrids (*Dryas octopetala x integrifolia*) (Philipp & Siegismund, 2003). Among the study plant species, Alpine bistort, Mountain avens, and Arctic willows associate with ectomycorrhizal fungi (Gardes & Dahlberg, 1996), Purple saxifrage with both arbuscular mycorrhizal fungi and with ectomycorrhizal fungi (Fujimura & Egger, 2012), and Moss champion with both ericoid mycorrhizal fungi (Kohn & Stasovski, 1990) and ectomycorrhizal fungi (Read & Haselwandter, 1981). Endophytic fungi are expected to be found in all five plant species (Strobel, 2018).

The sampling scheme was based on a hierarchical design, in which plant individuals were sampled within 18 locations (henceforth called sampling locations) across the western slope of the Aucella Mountain (Figure 1b). We selected 18 sampling locations that were roughly regularly spaced along the elevational gradient. The elevation of the sampling locations ranged from 33 to 479 m.a.s.l. (meters above sea level). At each location, we searched for the focal species by moving from a randomized starting point toward the South East along a 50‐m elevational isocline and collected root samples of five individuals of each of the five focal plant species. Different individuals of the same plant species were not selected if the distance between them was <1 m. The whole root system of the plant species was uprooted, and the samples consisted of the fine roots. The fine root samples were cleaned of soil particles by hand, first in the field and later in the laboratory under lens. The cleaned roots were wrapped with tissue paper and placed in a plastic bag with silica gel. In each sampling location, we measured soil pH (in soil–water suspension), percentage of soil water content (using a soil volumetric water content probe), the depth of the active soil layer (as the distance until the frozen horizon), and vegetation cover (visually estimating the cover percentage of all vascular plants in a 1 m × 1 m area). These measurements were taken at three points and averaged for each sampling location. The variation of these environmental variables along elevation is shown in Figure S3.

### 2.2 DNA extraction and sequencing

Root samples were weighed and the whole samples ground into fine powder by using a ball mill (Retsch Mixer Mill MM400). After grinding, 10 mg of each sample was used for DNA extraction. For samples of <10 mg of root material, the whole sample was used for DNA extraction. DNA was extracted using the NucleoSpin^®^ Plant II kit (Macherey‐Nagel).

We amplified the internal transcribed spacer region 2 (ITS2) using the forward primer fITS7 (GTGARTCATCGAATCTTTG) (Ihrmark et al., 2012) and the reverse primer ITS4 (TCCTCCGCTTATTGATATGC) (White, Bruns, Lee, & Taylor, 1990). The internal transcribed spacer (ITS) is one of the most widely sequenced DNA markers in fungal community analyses and has been selected as the universal genetic barcode for fungi (Schoch et al., 2012). We note, however, that the ITS2 is not a suitable marker for discriminating among arbuscular mycorrhizal taxa (Krüger, Krüger, Walker, Stockinger, & Schüßler, 2012) and that this group of root‐associated fungi is thus underrepresented in our data.

Prior to PCR amplification, all the DNA extracts were diluted to 0.5 ng/µl with ddH_2_O based on NanoDrop Lite (Thermo Scientific) measurements. PCR amplifications were performed as in Clemmensen, Ihrmark, Durling, and Lindahl (2016), using fITS7‐ and ITS4‐primers that were both tagged with 104 unique identification tags. PCRs were run in a total volume of 50 µl for 22–35 cycles. The number of cycles was adjusted on a sample‐by‐sample basis to yield weak to moderately strong bands on the agarose gel with approximately the same strength for all samples. PCR products were cleaned using the AMPure kit (Beckman Coulter), and DNA concentrations were determined using the Qubit dsDNA HS Assay Kit (Life Technologies). Altogether 450 of the original 450 samples were successfully amplified and pooled into six composite samples, which were then cleaned using the Cycle‐Pure Kit (Omega), and verified for quality on a Bioanalyzer (Agilent Tech). Pooled amplicon mixes were sequenced on a PacBio RS II system (Pacific Biosciences) at SciLifeLab (Uppsala, Sweden). The sequence data are available in the Dryad data repository (Abarenkov et al., 2018a).

### 2.3 Bioinformatics analyses

Sequences were analyzed using the SCATA pipeline (Sequence Clustering and Analysis of Tagged Amplicons, http://scata.mykopat.slu.se) (Ihrmark et al., 2012). Sequences were screened for tags and primer sequences, requiring 90% match with primer sequences. Sequences with a mean quality score lower than 20 or containing bases with a score lower than 3 were discarded. The remaining sequences were aligned pairwise, using USEARCH, and clustered into operational taxonomic units (OTUs) by single linkage clustering with 1.5% maximum distance allowed for sequences to enter clusters, homopolymers collapsed to 3 bp, mismatch penalty 1, gap open penalty 0, and gap extension penalty 1. Remaining singletons were removed.

Molecular species identification was conducted using the probabilistic taxonomical placement method Protax‐Fungi (Abarenkov et al., 2018b). This method quantifies the probabilities of all possible taxonomic placements for each query. As parameterization data, Protax‐Fungi used a fungal taxonomy derived from *Index Fungorum* (Index Fungorum, 2016) and a fungal reference database derived from UNITE, including reference sequences for all focal species (Kõljalg et al., 2013). The identification probabilities given by Protax‐Fungi account for several sources of uncertainty, such as the possibility that the reference sequences are mislabelled or that the species behind the environmental sequences are missing from the taxonomy.

The identified fungal taxa were classified as mycorrhizal or endophytic based on the FUNGuild database (Nguyen et al., 2016) combined with the expertise of the authors (NA and BL). Mycorrhizal species were mostly ectomycorrhizal, but also some ericoid mycorrhizal fungal species were included. We classified as endophytic fungi those fungal taxa which were known to live within plant tissues without causing disease symptoms (Bacon & White, 2000). Fungal species with uncertain interaction ecology, for which different data sources provided contrasting information, which could not be taxonomically assigned (i.e., no hit) or which belonged to some other guild than mycorrhizal or endophytic (e.g. lichenized or saprotrophic fungi), were grouped as “unclassified.” The taxonomic assignment of the OTUs as well as their classification as mycorrhizal or endophytic is provided in Table S1.

The data include in total 605,174 sequences, on average 1,366 sequences (median 1,240, min 105, max 19,146) for each of the 443 samples.

### 2.4 Statistical analyses

To describe general patterns in RAF community composition, we first applied a NMDS analysis to square root transformed data on relative OTU counts (number of reads for each OTU divided by the total number of reads in each sample) using the Bray–Curtis dissimilarity measure with the vegan R package (Oksanen et al., 2015). To explore how species richness (number of OTUs per plant individual) changed along the environmental gradient, we fitted generalized linear model assuming the log‐link function and the Poisson distribution, using the lme4 R package (Bates, Mächler, Bolker, & Walker, 2015). The species richness was modeled as a function of plant species (categorical variable), elevation (continuous variable), the interaction between plant species and elevation, soil pH (continuous), soil water content (continuous), depth of the active soil layer (continuous), and vegetation cover (continuous). As the occurrences of some RAF have been reported to peak at an intermediate elevation (Miyamoto et al., 2014), we additionally included the square of elevation. We controlled for variation in sequencing depth by including the log‐transformed total number of sequences for each sample as a continuous variable (Tedersoo et al., 2014). To account for the nonindependency of the samples acquired within the same sampling locations, the sampling location was included as a random effect. We selected the most parsimonious model following backward variable selection based on the Akaike Information Criterion (AIC) (Burnham & Anderson, 2002). We replicated the analyses for the full RAF community and for mycorrhizal, endophytic, and unclassified fungi separately.

Except for the species richness analyses described above, OTUs with <5% prevalence (i.e., occurring in <5% of the sampling units) were excluded from all analyses, leaving 231 OTUs. We replicated the analyses for the full RAF community (231 OTUs) and for mycorrhizal (78 OTUs), endophytic (63 OTUs), and unclassified fungi (90 OTUs) separately.

To quantify how much the host plant specialization varies between mycorrhizal and endophytic communities, and whether the level of specialization changes with elevation, we used a joint species distribution modeling approach (Ovaskainen et al., 2017; Warton et al., 2015). This approach allowed us to jointly model the occurrences and abundances of the fungal species identified in each plant individual (i.e., the whole interaction network) as a function of the environmental variables, while accounting for the structure of the study design. We quantified the network‐level specialization by quantifying the variance explained by the plant species in the joint species distribution models. We evaluated whether the network‐level specialization changed along elevation by adding an interaction term between plant species and elevation in the joint species distribution model. We fitted three alternative sets of hurdle‐type models and compared their performance. In these models, the presence–absences were modeled using a probit‐link function and the sequence counts were modeled using a log‐normal model conditional on presence. The compared alternative models were:

1. “no‐specialization model”—the first model assumes no specialization and therefore contains only following fixed effects: elevation (a continuous variable), the squared term of elevation, soil pH (continuous), soil water content (continuous), depth of the active soil layer (continuous) vegetation cover (continuous), and sequencing depth (log transformed, continuous);
2. “uniform specialization model”—the second model assumes uniform specialization, so it additionally includes the plant species as a categorical fixed effect;
3. “changing specialization model”—the third model assumes that the specialization changes with elevation, for which reason it features both the plant species term and the interaction term between plant species and elevation.

The sampling location was included as a random effect in all three alternative models. The models were fitted for the full RAF community and for mycorrhizal, endophytic and unclassified fungi separately.

We fitted all models using R package Hmsc 3.0 (Tikhonov et al., 2019) assuming the default prior distributions. The settings applied for posterior sampling through Markov chain Monte Carlo (MCMC) and the results of MCMC convergence are provided in the Appendix S1.

We assumed that there was evidence for specialization when the uniform specialization model performed better than the no‐specialization model. We assumed that there was evidence for changing specialization if the changing specialization model performed better than the uniform specialization model. We evaluated the model performances in two ways. First, we compared the models by the Widely Applicable Information Criterion (WAIC) (Watanabe, 2010), which gives an overall measure that evaluates the goodness of fit from the viewpoint of the entire community. Second, we compared the predictive powers of the models by a fivefold cross‐validation. For this, we split the five individuals of each plant species from each sampling location into five different folds. In this way, the fivefold cross‐validation should have maximum predictive power, as it has access to data on four plant individuals from the same sampling location to predict the fungal community in the fifth one. We measured predictive power separately for each species by AUC in the presence–absence model, and by R2 in the abundance (conditional on presence) model, as implemented in Tikhonov et al. (2019). We compared the models by their mean predictive power over the species, as well as the proportion of species for which one model yielded a higher predictive power than another model. The best performing models were those with the lowest absolute WAIC values and the highest predictive power in the fivefold cross‐validation.

To examine which variables were the most important in influencing fungal communities, we partitioned the explained variation among the explanatory variables for the best‐supported models.

## 3 RESULTS

In total, 2,874 RAF species (OTUs) were identified, out of which 695 were classified as mycorrhizal and 659 as endophytic fungi. The average number of fungal OTUs per plant individual was 57 (*SD* 10.4), with *Silene acaulis* having more RAF species (69 on average) than the other four plant species studied (51–58 on average) (Supporting Results, Figure S4). The number of mycorrhizal and endophytic fungi per plant individual was very similar, with *Silene acaulis* having the highest average for both mycorrhizal and endophytic fungal OTUs, and *Saxifraga oppositifolia* the least mycorrhizal OTUs (Supporting Results, Figure S4).

**FIGURE 2:**
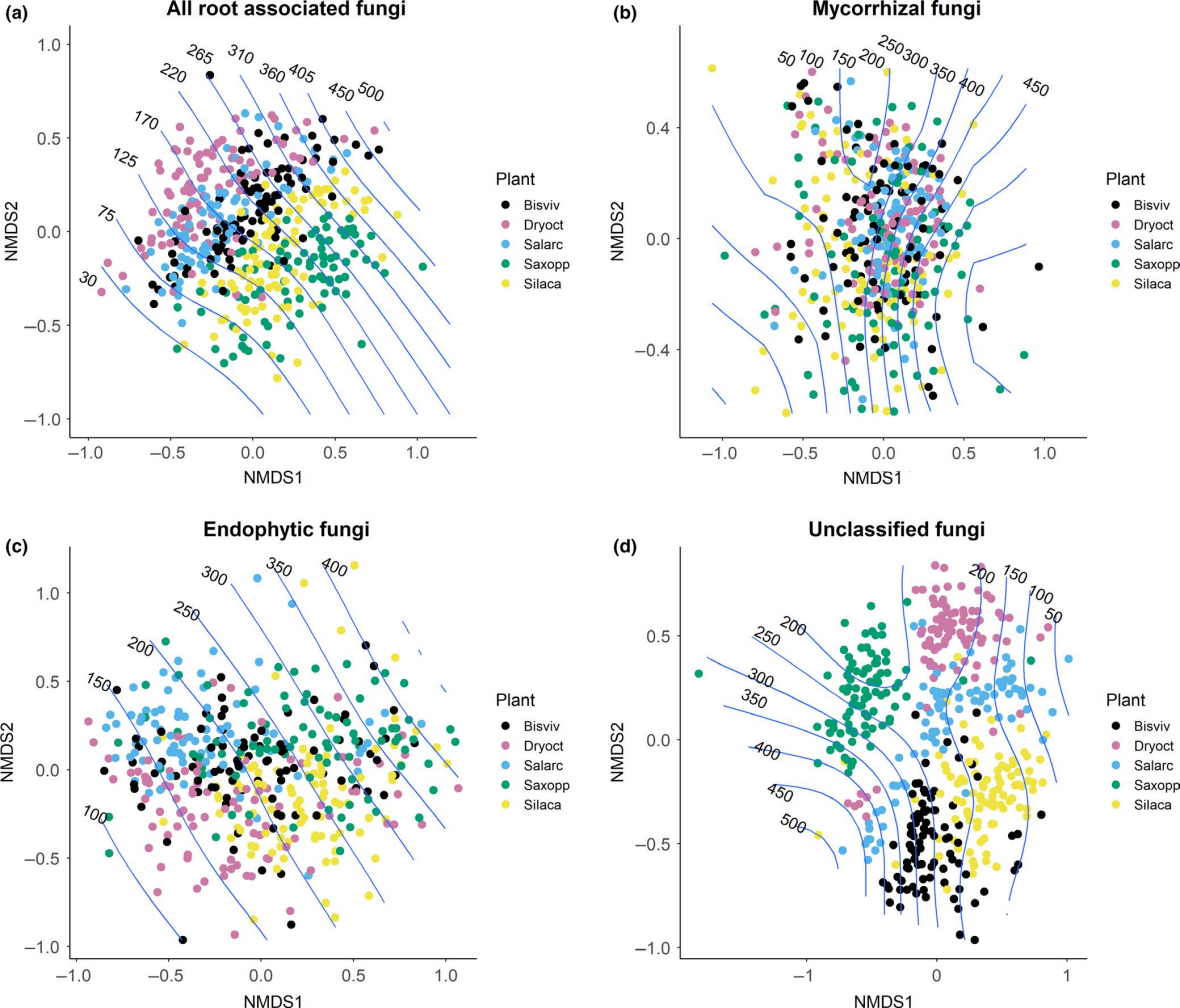
RAF species richness (number of OTUs) along elevation. Each dot shows the number of species in each sampling location, and the lines the predicted species richness. The colors represent the different plant species. The names of the five host plant species are abbreviated as follows: *Bistorta vivipara* as Bisviv*, Dryas octopetala x integrifolia* as Dryoct, *Salix arctica* as Salarc, *Saxifraga oppositifolia* as Saxopp, and *Silene acaulis* as Silaca

Root‐associated fungal OTU richness was influenced by both host plant identity and elevation. Yet, the effect of elevation varied among plant species, as shown by a significant interaction between plant species and elevation (Supporting Results, Table S2). Among all potential environmental variables considered, the most parsimonious models for the overall RAF community, endophytic fungi, and unclassified fungi included host plant identity, elevation, the square term of elevation, and the interaction term between elevation and plant species (Supporting Results, Table S2). The most parsimonious model for mycorrhizal OTU richness included the same set of variables, but lacked the interaction term between plant species and elevation. Overall RAF OTU richness decreased with elevation, especially in the case of *Saxifraga oppositifolia* (Figure 2a). The number of endophytic OTUs showed a slight peak at intermediate elevation, while for mycorrhizal fungi, the lowest OTU richness was found at the intermediate elevation (Figure 2b,c). The number of mycorrhizal OTUs associated with *Dryas octopetala x integrifolia* and *Salix arctica* increased slightly with elevation.

**FIGURE 3:**
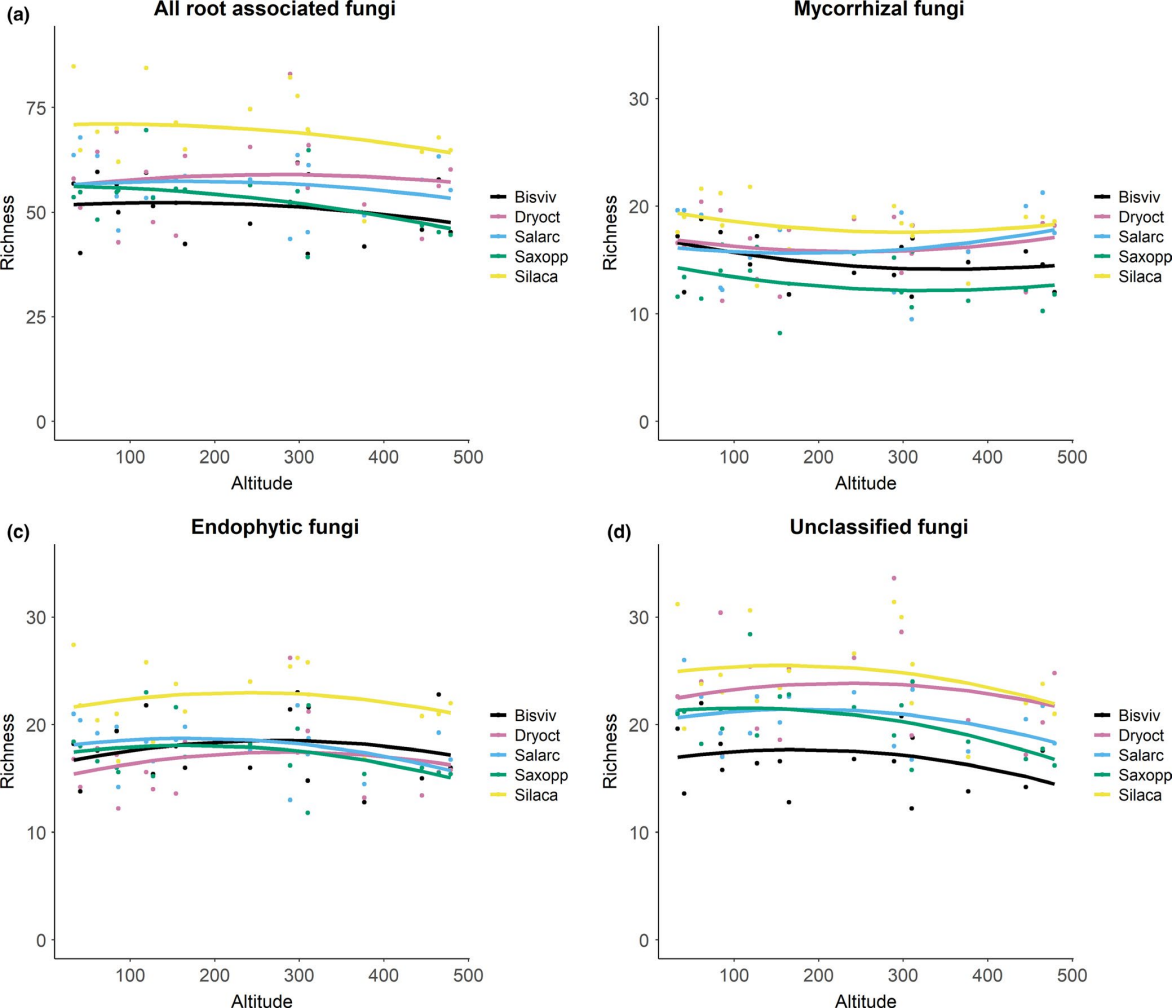
NMDS ordinations showing variation in RAF community composition in relation to elevation and host plant species. The plant species are indicated by different colors and the elevation by isoclines. Panel (a) shows the NMDS ordination applied to all root‐associated fungal species, Panel (b) to the subset of mycorrhizal species, Panel (c) to endophytic species, and Panel (d) to unclassified species. The names of the five host plant species are abbreviated as follows: *Bistorta vivipara* as Bisviv*, Dryas octopetala x integrifolia* as Dryoct, *Salix arctica* as Salarc, *Saxifraga oppositifolia* as Saxopp, and *Silene acaulis* as Silaca

The NMDS analysis showed that overall, RAF communities were structured by both the identity of the host plant species and elevation (Figure 3a, Stress level* = *0.1). This overall pattern was driven by the endophytic and the unclassified fungi, as the composition of mycorrhizal fungi was not structured by the identity of the plant species (Figure 3b,c,d).

The HMSC analyses showed that RAF communities were specialized on host plant species and that the degree of specialization was uniform along the elevational gradient. Model comparison based on WAIC showed that in all cases, the uniform specialization model performed better than the no‐specialization model (Table 1). The level of support for host specialization was lowest for mycorrhizal fungi, intermediate for endophytic fungi, and highest for the group of unclassified species. The model of changing specialization with elevation received only slightly lower WAIC values than the model of uniform specialization (Table 1) and was, thus, not conclusively supported. Model comparison based on predictive power provided results consistent with those from the WAIC comparison: There was support for host specialization, especially for the endophytic and unclassified groups, but little evidence of changing specialization along the elevation gradient (Table 1).

**TABLE 1:** Model comparison based on WAIC and predictive power

Fungal group	Data type	WAIC			Predictive power				
		m1	m2	m3	E[m1]	E[m2]	E[m3]	m2 > m1	m3 > m2
All RAF	Presence–absence	77.34	70.89*	70.89*	0.69	0.75*	0.75*	0.74*	0.38
	Abundance	56.36	54.25*	54.34	0.01	0.06*	0.06*	0.71*	0.46
Mycorrhizal	Presence–absence	23.76	23.64*	23.76	0.70	0.71*	0.70	0.62*	0.27
	Abundance	15.15	14.88*	14.93	−0.01	0.04*	0.04*	0.72*	0.50
Endophytic	Presence–absence	22.07	20.93*	21.00	0.70	0.75*	0.75*	0.84*	0.46
	Abundance	20.78	19.74*	19.79	−0.01	0.06*	0.06*	0.73*	0.46
Unclassified	Presence–absence	31.94	26.59*	26.65	0.67	0.79*	0.78	0.84*	0.42
	Abundance	20.65	19.74*	19.78	0.01	0.07*	0.07*	0.77*	0.44

Note

The values show the WAIC and predictive power calculated for the model assuming no specialization for the host plant species (m1), the model assuming uniform specialization along elevation (m2), and the model assuming changing specialization along elevation (m3). Predictive power is measured by AUC for presence–absence (P‐A) part and by *R* ^2^ for the abundance part of the hurdle‐model. The columns E[m1], E[m2], and E[m3] show the mean predictive power over the fungal species in models m1, m2, and m3. The columns m2 > m1 and m3 > m2 show the proportion of species for which predictive power is higher in model m2 than in model m1, or higher in model 3 than in model 2. The best model fit is indicated with an asterisk, as identified by the lowest WAIC value, the highest mean predictive, and more than half of the species having a higher predictive power in the focal model compared to an alternative simpler model.

In the same line, the comparison among the species‐specific specialization values showed that the unclassified species were the among most specialists followed by endophytic species and mycorrhizal species (Table 2). For example, the most specialized unclassified species (scata3357_3; specialization level 0.51) had prevalence 1 in *Saxifraga oppositifolia* (meaning that it occurred in all *Saxifraga oppositifolia* individuals in the data), whereas its prevalence in the other plant species ranged from 0 to 0.04 (Table 2). In comparison, the most specialized endophytic species (scata3357_83, Sordariales; specialization level 0.21) had prevalence 0.64 in *Salix arctica*, and its prevalence ranged from 0.08 to 0.22 in the other plant species and the most specialized mycorrhizal species (scata3357_362, Sebacinaceae; specialization level 0.18) had prevalence 0.15 in *Salix arctica* and its prevalence ranged from 0 to 0.07 in the other plant species.

**TABLE 2:** Plant‐specific prevalences for the ten most specialized species

Mycorrhizal fungi OTU	Protax‐fungi identification	AUC(m2)‐AUC(m1)	*Bistorta vivipara*	*Dryas octopetala*	*Salix arctica*	*Saxifraga oppositifolia*	*Silene acaulis*
scata3357_362	Sebacinaceae	0.18	0.07	0.04	0.15	0.00	0.00
scata3357_5	Gloniaceae	0.09	0.93	0.94	0.96	0.75	0.88
scata3357_182	Thelephoraceae	0.08	0.04	0.04	0.06	0.02	0.13
scata3357_308	Thelephoraceae	0.06	0.04	0.06	0.12	0.00	0.07
scata3357_1513	Cortinariaceae	0.06	0.26	0.21	0.27	0.31	0.51
scata3357_128	Thelephoraceae	0.05	0.08	0.22	0.14	0.07	0.18
scata3357_178	Inocybaceae	0.04	0.04	0.09	0.13	0.04	0.20
scata3357_198	Sebacinaceae	0.04	0.06	0.11	0.01	0.04	0.10
scata3357_93	Cortinariaceae	0.04	0.08	0.08	0.10	0.09	0.24
scata3357_41	Cortinariaceae	0.03	0.41	0.40	0.25	0.22	0.38

Endophytic fungi OTU	Protax‐fungi identification	AUC(m2)‐AUC(m1)	*Bistorta vivipara*	*Dryas octopetala*	*Salix arctica*	*Saxifraga oppositifolia*	*Silene acaulis*
scata3357_83	Sordariales	0.21	0.14	0.09	0.64	0.08	0.22
scata3357_153	Helotiales	0.18	0.26	0.14	0.56	0.01	0.09
scata3357_259	Helotiales	0.16	0.09	0.08	0.29	0.03	0.01
scata3357_272	Chaetothyriales	0.14	0.08	0.02	0.02	0.22	0.07
scata3357_27	Helotiales	0.12	0.78	0.33	0.43	0.38	0.68
scata3357_399	Capnodiales	0.12	0.00	0.14	0.04	0.08	0.10
scata3357_531	Chaetothyriales	0.12	0.04	0.09	0.04	0.04	0.19
scata3357_527	Chaetothyriales	0.11	0.04	0.07	0.04	0.02	0.29
scata3357_630	Chaetothyriales	0.11	0.03	0.09	0.00	0.04	0.14
scata3357_136	Helotiales	0.11	0.08	0.00	0.05	0.09	0.17

Unclassified fungi OTU	Protax‐fungi identification	AUC(m2)‐AUC(m1)	*Bistorta vivipara*	*Dryas octopetala*	*Salix arctica*	*Saxifraga oppositifolia*	*Silene acaulis*
scata3357_3	No hit	0.51	0.01	0.00	0.00	1.00	0.04
scata3357_16	No hit	0.47	0.00	0.00	0.01	0.00	0.98
scata3357_20	No hit	0.47	0.02	0.00	0.00	0.61	0.00
scata3357_13	No hit	0.43	0.03	0.98	0.04	0.02	0.02
scata3357_15	No hit	0.43	1.00	0.03	0.02	0.02	0.03
scata3357_469	No hit	0.41	0.00	0.00	0.00	0.44	0.01
scata3357_184	No hit	0.41	0.01	0.00	0.01	0.02	0.56
scata3357_17	No hit	0.40	0.02	0.00	1.00	0.03	0.01
scata3357_902	No hit	0.40	0.00	0.00	0.00	0.28	0.00
scata3357_189	No hit	0.35	0.02	0.41	0.00	0.00	0.01

Note

AUC(m2)‐AUC(m1) is a measure of specialization calculated as the difference in predictive power between model m2 that includes host plant specialization and model m1 that excludes host plant specialization. The remaining columns show the host plant‐specific prevalences, that is, the proportion of plant individuals out of which the focal species was found.

**FIGURE 4:**
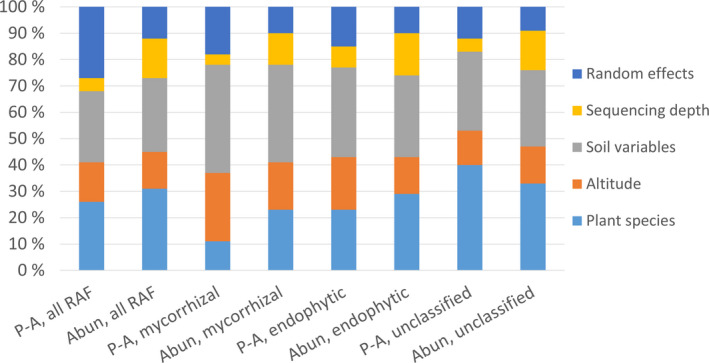
Community‐level variation partitioning for the models assuming uniform specialization along elevation. The amount of variance explained by each variable is the proportion of variance explained over the explanatory power of their corresponding models. The models have been fitted to the presence–absence (P‐A) and abundance conditional on presence (Abun) data. Elevation includes its linear effect as well as its unimodal effect (squared term), and soil variables include soil pH, soil water content, depth of the active soil layer, and vegetation cover. The random effect corresponds to the sampling location. The numerical values are provided in the Table S3

The best‐supported models (i.e., those assuming uniform specialization along elevation) indicated that the plant species explained a substantial amount of variation in RAF communities (Figure 4). This was especially the case for endophytic fungi and unclassified fungi, for which the plant species explained as much of the variance as all measured soil variables together (Figure 4, Table S3). In the case of mycorrhizal fungi, however, the soil variables explained more variation than the plant species (Figure 4, Table S3).

## 4 DISCUSSION

Our study revealed three main patterns of host associations of arctic root‐associated fungi which were in contrast to our expectations based on earlier studies (Botnen et al., 2014; Ryberg et al., 2011; Timling et al., 2012; Walker et al., 2011). First, while we expected a low degree of host specialization in the root‐associated fungal community in general, we found a clear signal of specialization on host plant species. However, we note that earlier studies have mostly focused on mycorrhizal communities (Botnen et al., 2014; Ryberg et al., 2011; Timling et al., 2012), and our results confirmed a low level of specialization in arctic mycorrhizal fungi. Second, we found endophytic fungi to be more specialized than the mycorrhizal fungi, and intriguingly, the most specialized to be those fungi that we could not classify to either group. Third, we expected RAF communities to be more generalist in terms of host associations at higher than lower elevations, as a more opportunistic strategy would help plants survive in nutrient‐poor environments (Botnen et al., 2014; Ryberg et al., 2011; Timling et al., 2012). Rather, we found RAF communities at higher elevations to be different and less species rich, but that each fungal species was equally specialized in terms of its host plant association along the altitudinal gradient. We next discuss each of these findings in turn.

Unlike mycorrhizal fungi, endophytic fungi can colonize plant parts other than roots and are generally considered opportunistic colonizers (Knapp et al., 2012; Mandyam et al., 2012), leading to our a priori assumption that they would be less specific to certain hosts. Contrarily, we found that endophytic fungi were more specialized on the host plant species than mycorrhizal fungi. Whether this pattern is characteristic of arctic ecosystems remains to be established. In temperate environments where mycorrhizal fungi are considered to be more specialist than in the Arctic, Toju et al. (2013) did not find differences in the species‐level specialization patterns between root‐associated endophytic and mycorrhizal taxa. Likewise, Põlme et al. (2018) assessed the host specialization patterns of endophytic and mycorrhizal fungi from data mainly collected in temperate and boreal ecosystems, and did not find differences between these two groups. Another possibility is that the fungi classified as endophytic are parasitic rather than mutualists, in which case greater specialization would have been expected (Borowicz & Juliano, 1991). In parasitic fungi, increasing host specialization has been suggested to be an adaptation to secure physiological compatibility with their hosts (Antonovics et al., 2013).

We found a high specialization on arctic root‐associated fungal endophytes, which was unexpected based on previous results. Notably, Walker et al. (2011) found no signs of specialization on arctic endophytic communities associated with roots of Ericaceae plants. The reason for the difference between our results and those by Walker et al. (2011) most likely reflects the phylogenetic distances of the studied host plant species. Walker et al. (2011) targeted host plant species belonging to the same family, while the five focal host plant species from our study represent five different families. Host phylogenetic distance strongly affects the root‐associated fungal communities, closely related hosts associating with more similar communities (Tedersoo, Mett, Ishida, & Bahram, 2013).

Low specialization by mycorrhizal fungi on the plant species of the Arctic has been suggested to be an adaptive response to low nutrient availability. Arctic plants may have established mycorrhizal partnerships with a wider range of fungi, as a way of ensuring nutrient uptake in these nutrient‐poor environments (Botnen et al., 2014; Ryberg et al., 2011; Timling et al., 2012). Although both mycorrhizal and endophytic fungi may have positive effects on plant fitness, the way in which they colonize plant roots are different. The plants and mycorrhizal fungi communicate chemically during the root colonization process, and to some extent, plants are able to regulate their level of mycorrhizal colonization (van der Heijden, Martin, Selosse, & Sanders, 2015). Concerning endophytic fungi, plants may lack the ability to regulate the symbiotic interplay (Kothe & Turnau, 2018). The notion that plants can regulate the range of mycorrhizal and endophytic fungi differently remains to be conclusively validated by experimental approaches.

Our results showed that host plants associated with a different set of RAF at higher elevations, but the range of species with which they associate remained constant, namely there was no evidence of changing specialization. Interestingly, this result contradicts the recent findings in fungal leaf endophytic communities whose host specialization varies with altitude (Cobian et al., 2019). This result supports the idea that although fungal endophytes can potentially colonize different plant tissues, there is little transmission of endophytes through plant tissues (Wearn et al., 2012). Therefore, fungal endophytic communities inhabiting different plant tissues may follow independent dynamics.

Changes in RAF community composition along elevational gradients have mostly been attributed to changes in the host plant distributions and abiotic conditions such as thinning of soil depth, limited nutrient availability, and colder climatic conditions at higher elevations (Bahram et al., 2012; Jarvis et al., 2015; Kivlin et al., 2017; Matsuoka et al., 2016; Miyamoto et al., 2014). The RAF community composition changes along elevation have been suggested to have important consequences for the success of plant seedling growth at higher elevations (Defossez et al., 2011; Wagg, Husband, Green, Massicotte, & Peterson, 2011). Our current findings largely match those of Jarvis et al. (2015), who found that species richness changed little but community varied strongly along elevation. In another study focusing on variation in ectomycorrhizal fungal richness along elevation, Miyamoto et al. (2014) found that species richness peaked at mid elevations, due to a mid‐domain effect in the distributional ranges of the ectomycorrhizal species. Unlike our approach, Miyamoto et al. (2014) did not sample the RAF associated with the same host species along the whole elevational gradient, but acquired soil core samples. Clearly, such samples will represent the roots of as many species as were present at the sampling sites, combining the signal from host associations with turnover in the pool of plant species.

Understanding of fungal communities from a functional perspective is currently expanding, not least as a result of increasing taxonomic coverage of reference sequence databases (Abarenkov et al., 2010; Nilsson et al., 2018) and ecological annotation platforms, which allow ecologists to link molecularly identified species to their ecological roles (Nguyen et al., 2016). In our study, there were still many fungal OTUs which could not be assigned to a fungal taxa and/or functional group, or even they were assigned to fungal taxa which do not interact with plant roots (e.g., lichenized fungi or saprotrophs). While some of the latte species might have popped up due to soil contamination in the root samples, this result also reflects a major knowledge gap concerning arctic fungi. Compared to other groups or organisms, the reference databases for fungi are largely incomplete or mislabeled, which leads to erroneous taxonomic assignments (Somervuo et al., 2017). Curiously, the unclassified fungi showed the highest level of specialization for the host plant species. The most abundant species which were grouped as unclassified fungi are species from the genus *Mycena*. Although *Mycena* species have traditionally been considered saprotrophs, there is growing evidence that they facultatively colonize plant roots (Grelet et al., 2017).

While targeting both endophytic and mycorrhizal fungi, our paper leaves other important organism groups unresolved. Two types of root‐associated organisms of specific global importance are arbuscular mycorrhizal fungi (Öpik, Moora, Liira, & Zobel, 2006; Öpik et al., 2013) and root‐associated bacteria (Fierer & Jackson, 2006). In our study, basically all species classified as mycorrhizal were explicitly ectomycorrhizal, since to efficiently detect arbuscular mycorrhizal fungi, a different set of primers is needed (Lekberg et al., 2018). Assessing the specialization levels of such additional root‐associated organisms in the Arctic would be an exciting research avenue. In particular, it would be interesting to assess whether the host specialization of arbuscular mycorrhizal fungi differs from that found in other ecosystems, that is, whether the specialization level of arbuscular mycorrhizal species in the Arctic is lower than in other ecosystems, as we found to be the case for (ecto)mycorrhizal fungi.

Overall, our study suggested that host plant identity affects mycorrhizal and endophytic fungi differently. Whether these findings are unique to the high arctic realm, or whether our findings point to broader patterns across biomes, remains to be solved by future studies. In particular, it would be highly insightful to compare the specialization level of mycorrhizal and endophytic fungi on the same set of host plants species across types of ecosystems. We hope that our study may serve as a template for such future studies.

## CONFLICT OF INTEREST

We declare no conflict of interest.

## AUTHOR CONTRIBUTION

**Nerea Abrego:** Conceptualization (lead); Data curation (lead); Formal analysis (lead); Investigation (lead); Methodology (lead); Writing‐original draft (lead); Writing‐review & editing (lead). **Tea Huotari:** Data curation (lead); Investigation (lead); Methodology (lead); Writing‐original draft (equal); Writing‐review & editing (equal). **Ayco J. M. Tack:** Conceptualization (equal); Funding acquisition (equal); Investigation (equal); Project administration (equal); Writing‐original draft (equal); Writing‐review & editing (equal). **Björn D. Lindahl:** Conceptualization (equal); Data curation (equal); Methodology (equal); Writing‐original draft (equal); Writing‐review & editing (equal). **Gleb Tikhonov:** Formal analysis (equal); Methodology (equal). **Panu Somervuo:** Data curation (equal); Methodology (equal). **Niels Martin Schmidt:** Funding acquisition (equal); Project administration (equal); Writing‐original draft (equal); Writing‐review & editing (equal). **Otso Ovaskainen:** Conceptualization (equal); Formal analysis (equal); Methodology (equal); Writing‐original draft (equal); Writing‐review & editing (equal). **Tomas Roslin:** Conceptualization (equal); Funding acquisition (equal); Investigation (equal); Project administration (equal); Supervision (equal); Writing‐original draft (equal); Writing‐review & editing (equal).

## Supporting information

Appendix S1

## Data Availability

The original sequence data are available in the Dryad data repository (https://doi.org/10.5061/dryad.9dr6j0c), and the molecular species identifications are provided as Table S1.
