# Supplementary material for: Higher host plant specialization of root‐associated endophytes than mycorrhizal fungi along an arctic elevational gradient
Source: Ecol Evol. 2020 Aug 6;10(16):8989–9002. doi: 10.1002/ece3.6604 (PMC7452766; doi:10.1002/ece3.6604)
Supplement: Supplementary file 1 — Appendix S1 [file ECE3-10-8989-s001.pdf]

Higher host-plant specialization of root-associated endophytes than mycorrhizal fungi along an arctic elevational gradient

Nerea Abrego, Tea Huotari, Ayco J. M. Tack, Björn D. Lindahl, Gleb Tikhonov Panu Somervuo, Niels Martin Schmidt, Otso Ovaskainen & Tomas Roslin

SUPPLEMENTARY MATERIALS AND METHODS

Convergence of the Markov chain Monte Carlo (MCMC) scheme used for posterior sampling

We obtained for each model 1000 samples for each of the four MCMC chains, and thus in total 4000 samples each. The 1000 samples for each chain were obtained by running the MCMC for 1500*thin iterations, out of which 500*thin were discarded as transient, and the remaining 1000*thin iterations were then evenly thinned to yield 1000 samples. We applied thin=1, 10, 100 unless the convergence was satisfactory or unless the computations took >1 week for each model (fitting the models to data on all fungi was computationally much more intensive than for the subsets of species). We assessed MCMC convergence by computing the effective number of samples and the potential scale reduction factor. These are shown in Fig. S3 (effective number of samples) and Fig. S4 (potential scale reduction factors) for the beta-parameters of the HMSC model (Tikhonov et al. 2019), which parameters measure the responses of the species to the included covariates, and thus on which the results of the paper are predominantly based on. As the effective number of samples are high (typically close to 4000, i.e. the actually number of samples) and the potential scale reduction factors are close to one, we conclude the MCMC convergence was satisfactory.

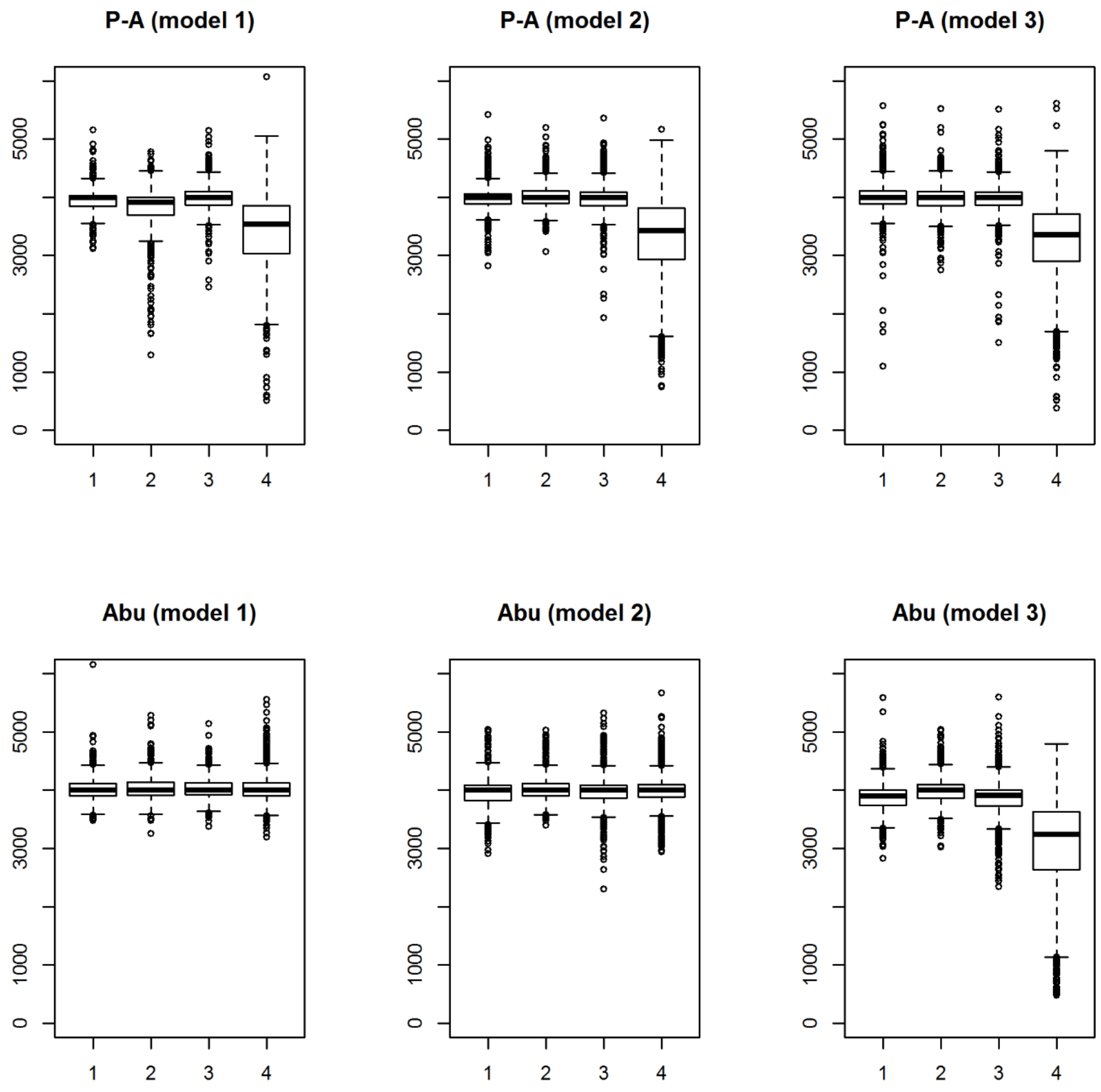

Figure S1. Effective number of posterior samples obtained by the MCMC scheme. The upper row of panels corresponds to the presence-absence models and the low row of panels to the abundance models. The columns correspond to model 1 (no specialization), model 2 (uniform specialization), and model 3 (changing specialization). In each panel, the results are shown for mycorrhizal species (1), endophytic species (2), unclassified species (3), and all RAF (4). Each boxplot shows the distribution of effective number of posterior samples over the species-specific beta-parameters included in the model.

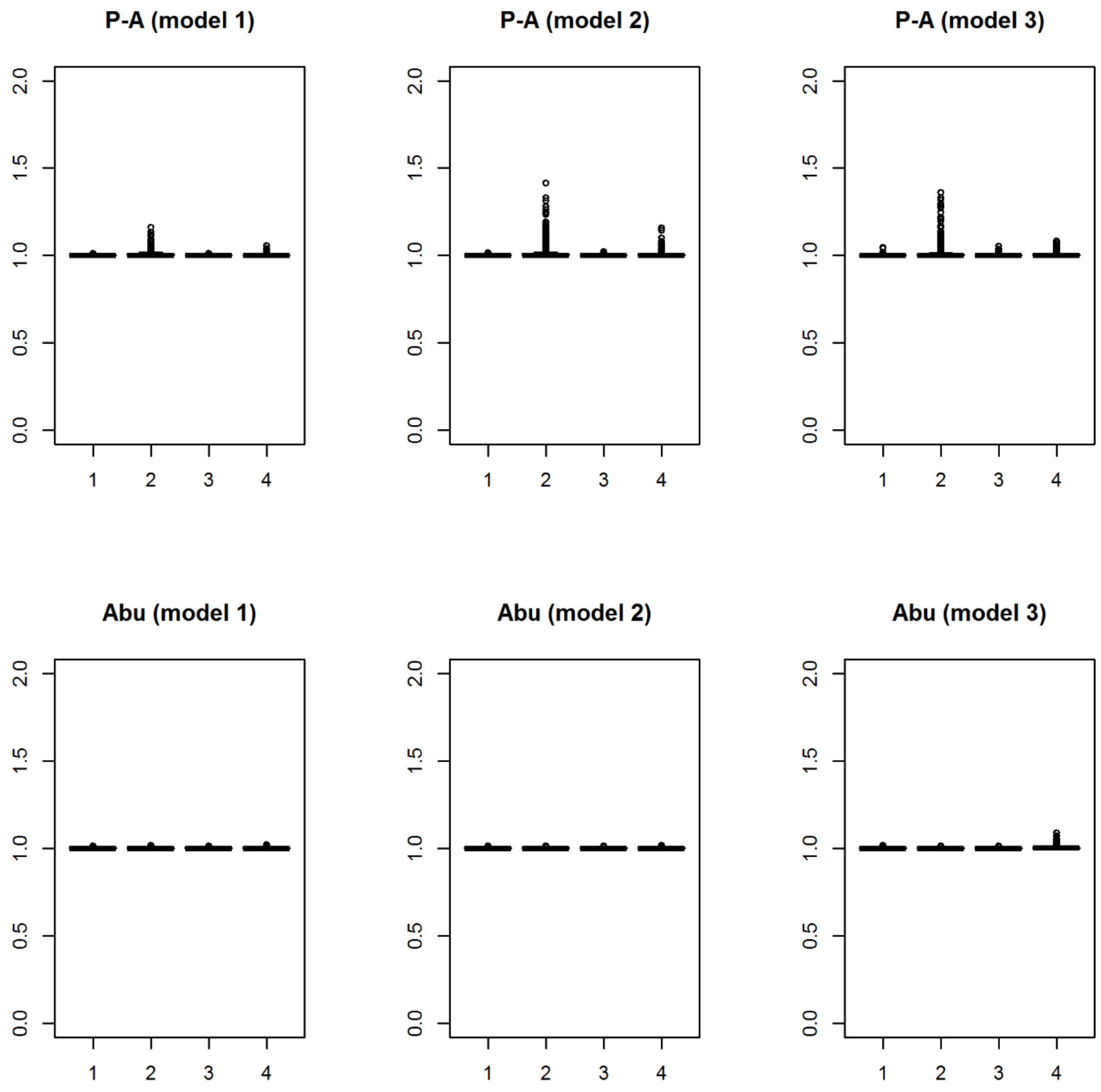

Figure S2. Potential scale reduction factors of the posterior samples obtained by the MCMC scheme. The upper row of panels corresponds to the presence-absence models and the low row of panels to the abundance models. The columns correspond to model 1 (no specialization), model 2 (uniform specialization), and model 3 (changing specialization). In each panel, the results are shown for mycorrhizal species (1), endophytic species (2), unclassified species (3), and all RAF (4). Each boxplot shows the distribution of potential scale reduction factors over the species-specific beta-parameters included in the model.

SUPPLEMENTARY RESULTS

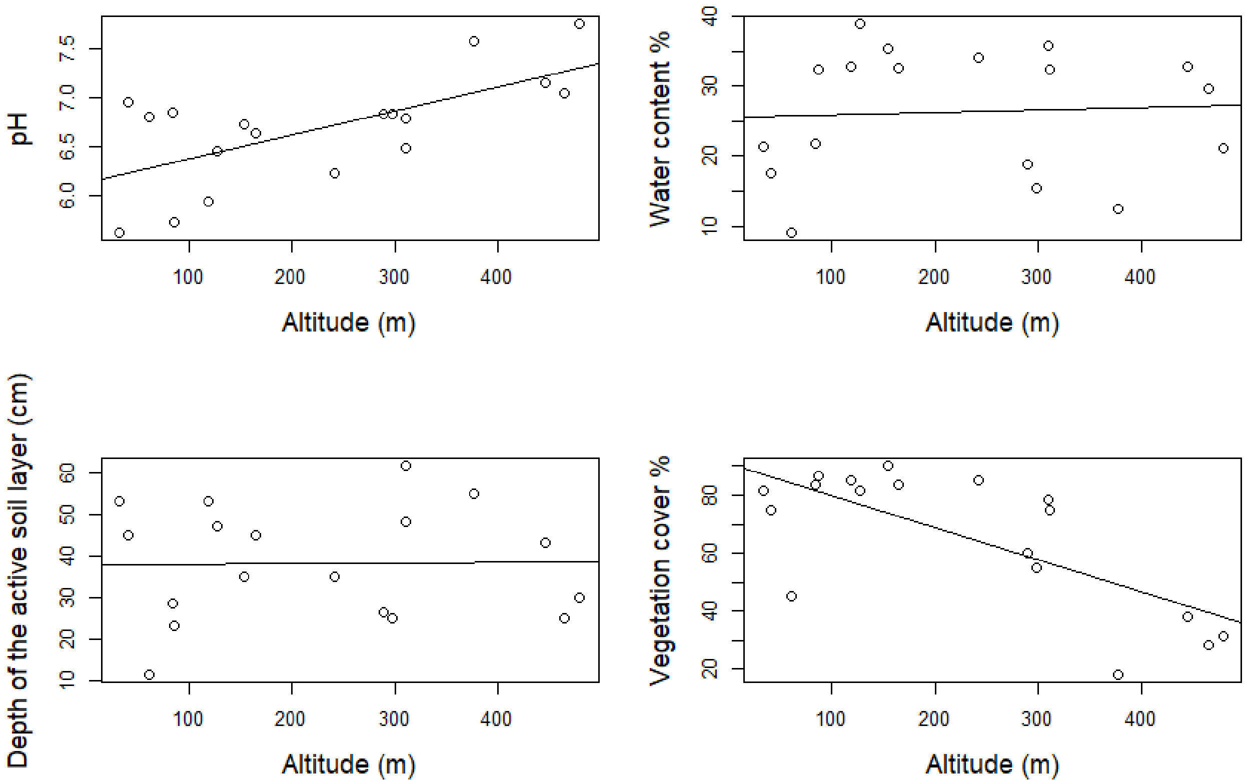

Figure S3. Changes in environmental conditions with elevation. Each data point refers to an individual sampling location.

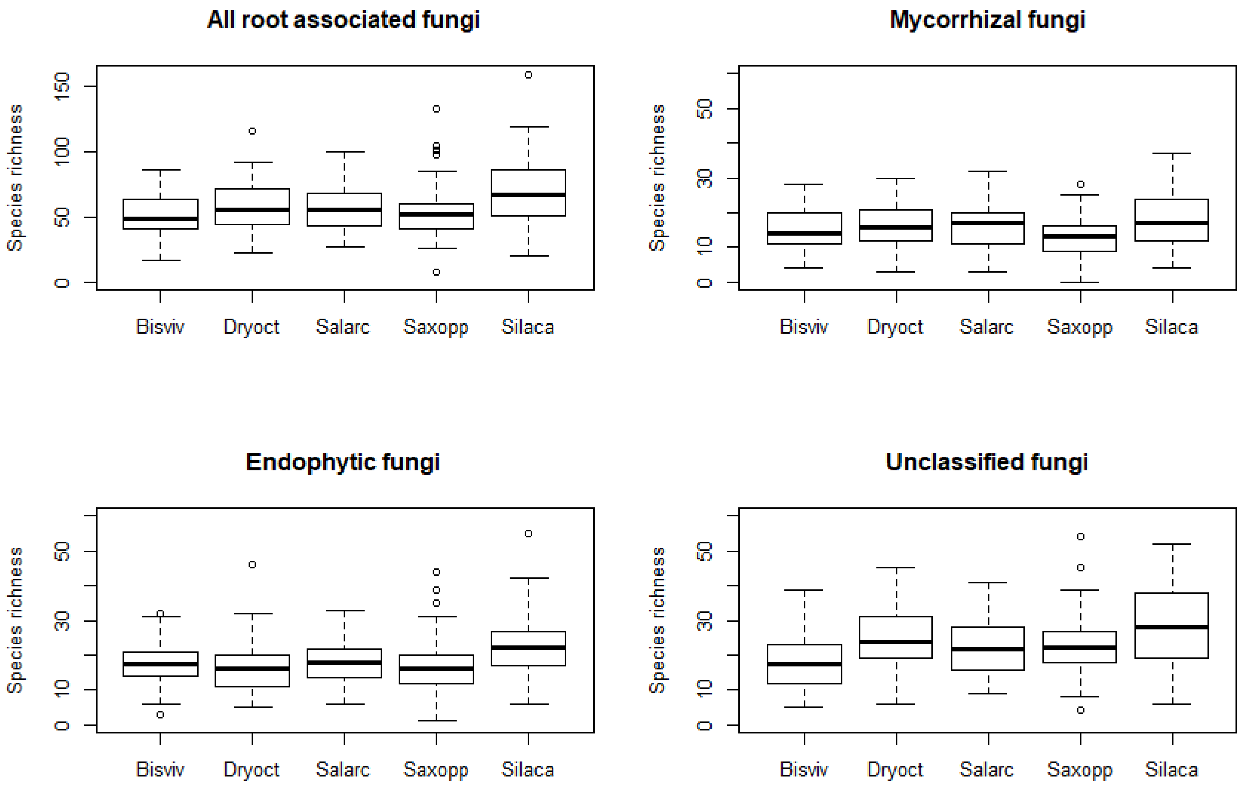

Figure S4. Species richness per plant individual. Panel A shows the species richness of all root associated fungal species, Panel B of the subset of mycorrhizal species, Panel C of endophytic species, and Panel D of unclassified species. The names of the five host plant species are abbreviated as follows: *Bistorta vivipara* as Bisviv, *Dryas octopetala x integrifolia* as Dryoct, *Salix arctica* as Salarc, *Saxifraga oppositifolia* as Saxopp and *Silene acaulis* as Silaca.

Table S1. OTU classification used in this study. The first sheet shows the Scata OTU cluster ids, and their classification as mycorrhizal and endophytic fungi. The cases in which different sequences from the same cluster yielded different species assignments are classified as “mixed”, the cases which could not be assigned to any taxa are classified as “no hit” and the cases for which the assigned taxa belong to some other group than mycorrhizal or endophytic (e.g. lichenized fungi and saprotrophic fungi) are classified as “other”.

OTU	Classification
scata3357_1	Endophyte
scata3357_0	Endophyte
scata3357_5	Mycorrhizal
scata3357_2	Endophyte
scata3357_4	Endophyte
scata3357_3	No hit
scata3357_6	Other
scata3357_10	Mycorrhizal
scata3357_8	No hit
scata3357_7	Mycorrhizal
scata3357_9	Other
scata3357_12	No hit
scata3357_11	Mixed
scata3357_18	Endophyte
scata3357_20	Other
scata3357_22	Endophyte
scata3357_29	Other
scata3357_13	No hit
scata3357_14	Other
scata3357_19	No hit
scata3357_17	No hit
scata3357_15	No hit
scata3357_24	Endophyte
scata3357_16	No hit
scata3357_27	Endophyte
scata3357_37	Endophyte
scata3357_38	Endophyte
scata3357_31	Mixed
scata3357_48	Endophyte
scata3357_21	Mycorrhizal
scata3357_25	Mycorrhizal
scata3357_33	Mycorrhizal
scata3357_30	Mycorrhizal
scata3357_26	Mycorrhizal
scata3357_60	Endophyte
scata3357_23	Other
scata3357_28	Mycorrhizal
scata3357_42	Endophyte
scata3357_34	Other
scata3357_36	No hit
scata3357_32	Mycorrhizal
scata3357_41	Mycorrhizal
scata3357_45	No hit
scata3357_65	Mycorrhizal
scata3357_39	Mycorrhizal
scata3357_40	Endophyte
scata3357_35	Mycorrhizal
scata3357_58	Endophyte
scata3357_66	Endophyte
scata3357_49	Other
scata3357_50	No hit
scata3357_56	No hit
scata3357_79	No hit
scata3357_69	No hit
scata3357_51	Mycorrhizal
scata3357_53	Mycorrhizal
scata3357_70	Endophyte
scata3357_44	No hit
scata3357_61	Mycorrhizal
scata3357_52	Mycorrhizal
scata3357_64	Mixed
scata3357_43	Mycorrhizal
scata3357_54	Endophyte
scata3357_63	Mycorrhizal
scata3357_57	Endophyte
scata3357_71	No hit
scata3357_73	Mycorrhizal
scata3357_81	Other
scata3357_62	No hit
scata3357_76	Mixed
scata3357_86	No hit
scata3357_47	Mycorrhizal
scata3357_46	No hit
scata3357_96	Endophyte
scata3357_80	Other
scata3357_68	Mycorrhizal
scata3357_59	Mycorrhizal
scata3357_77	No hit
scata3357_87	Mycorrhizal
scata3357_83	Endophyte
scata3357_99	Endophyte
scata3357_72	Mycorrhizal
scata3357_101	Other
scata3357_94	No hit
scata3357_74	Mixed
scata3357_90	Other
scata3357_100	No hit
scata3357_98	Mycorrhizal
scata3357_119	Other
scata3357_84	Other
scata3357_93	Mycorrhizal

scata3357_82	Mycorrhizal
scata3357_118	Other
scata3357_108	Endophyte
scata3357_121	Mycorrhizal
scata3357_104	Endophyte
scata3357_75	Mycorrhizal
scata3357_91	Mycorrhizal
scata3357_85	Mycorrhizal
scata3357_78	Other
scata3357_95	Mycorrhizal
scata3357_130	Mixed
scata3357_88	Mycorrhizal
scata3357_146	No hit
scata3357_106	Endophyte
scata3357_107	Mycorrhizal
scata3357_124	No hit
scata3357_112	Mycorrhizal
scata3357_131	Endophyte
scata3357_115	Mycorrhizal
scata3357_138	Endophyte
scata3357_102	Mycorrhizal
scata3357_148	Endophyte
scata3357_144	Other
scata3357_105	Mycorrhizal
scata3357_113	Mycorrhizal
scata3357_97	Mycorrhizal
scata3357_103	Mycorrhizal
scata3357_120	Mycorrhizal
scata3357_109	Mycorrhizal
scata3357_125	Mycorrhizal
scata3357_136	Endophyte
scata3357_92	Mycorrhizal
scata3357_111	Other
scata3357_126	Mycorrhizal
scata3357_117	Mycorrhizal
scata3357_122	Mycorrhizal
scata3357_158	No hit
scata3357_114	Mycorrhizal
scata3357_153	Endophyte
scata3357_150	Other
scata3357_143	Mixed
scata3357_116	Mycorrhizal
scata3357_164	No hit
scata3357_140	Mycorrhizal
scata3357_147	Endophyte
scata3357_141	No hit
scata3357_151	Mycorrhizal
scata3357_128	Mycorrhizal
scata3357_129	Mycorrhizal
scata3357_110	Other
scata3357_133	Mycorrhizal
scata3357_170	Other
scata3357_135	Mycorrhizal

scata3357_155	No hit
scata3357_123	Mycorrhizal
scata3357_139	Mycorrhizal
scata3357_189	No hit
scata3357_149	Mycorrhizal
scata3357_137	Mycorrhizal
scata3357_160	No hit
scata3357_201	Mycorrhizal
scata3357_195	No hit
scata3357_134	Mycorrhizal
scata3357_156	Mycorrhizal
scata3357_157	No hit
scata3357_166	Endophyte
scata3357_132	Mycorrhizal
scata3357_142	Mycorrhizal
scata3357_145	Mycorrhizal
scata3357_185	Endophyte
scata3357_159	Mycorrhizal
scata3357_163	Mycorrhizal
scata3357_165	Endophyte
scata3357_167	Mycorrhizal
scata3357_224	Endophyte
scata3357_177	Mycorrhizal
scata3357_171	Mycorrhizal
scata3357_188	Endophyte
scata3357_211	Other
scata3357_187	Mycorrhizal
scata3357_207	Endophyte
scata3357_173	Mycorrhizal
scata3357_202	No hit
scata3357_152	Mycorrhizal
scata3357_154	Other
scata3357_178	Mycorrhizal
scata3357_184	No hit
scata3357_186	Mycorrhizal
scata3357_175	Other
scata3357_168	Mycorrhizal
scata3357_210	Other
scata3357_174	Mycorrhizal
scata3357_182	Mycorrhizal
scata3357_179	No hit
scata3357_223	No hit
scata3357_162	Mycorrhizal
scata3357_193	Mycorrhizal
scata3357_203	Endophyte
scata3357_181	Mixed
scata3357_161	Mycorrhizal
scata3357_192	Mycorrhizal
scata3357_194	Mycorrhizal
scata3357_196	Mycorrhizal
scata3357_180	Mycorrhizal
scata3357_209	Mycorrhizal
scata3357_222	Endophyte

scata3357_248	Mixed
scata3357_254	Endophyte
scata3357_176	Mycorrhizal
scata3357_221	Other
scata3357_204	Endophyte
scata3357_239	Endophyte
scata3357_199	Mycorrhizal
scata3357_191	Mycorrhizal
scata3357_198	Mycorrhizal
scata3357_247	No hit
scata3357_229	Mycorrhizal
scata3357_242	Mycorrhizal
scata3357_214	Mycorrhizal
scata3357_287	Mixed
scata3357_190	Other
scata3357_197	Mycorrhizal
scata3357_183	Mycorrhizal
scata3357_220	Mycorrhizal
scata3357_262	Other
scata3357_216	No hit
scata3357_217	Endophyte
scata3357_227	No hit
scata3357_241	Endophyte
scata3357_208	Other
scata3357_205	Mycorrhizal
scata3357_213	Other
scata3357_218	Mycorrhizal
scata3357_215	Other
scata3357_240	Mycorrhizal
scata3357_289	No hit
scata3357_253	Other
scata3357_236	Other
scata3357_206	Mycorrhizal
scata3357_245	Endophyte
scata3357_266	Endophyte
scata3357_256	Endophyte
scata3357_258	Endophyte
scata3357_246	Endophyte
scata3357_255	Mixed
scata3357_225	Mycorrhizal
scata3357_237	Mycorrhizal
scata3357_212	Other
scata3357_235	Mycorrhizal
scata3357_226	Mycorrhizal
scata3357_273	Mycorrhizal
scata3357_260	Mixed
scata3357_250	Mycorrhizal
scata3357_252	Mixed
scata3357_238	Mycorrhizal
scata3357_277	Other
scata3357_232	Mycorrhizal
scata3357_243	Other
scata3357_271	Endophyte

scata3357_286	Endophyte
scata3357_251	Mycorrhizal
scata3357_269	Other
scata3357_322	Mycorrhizal
scata3357_234	Mycorrhizal
scata3357_259	Endophyte
scata3357_278	Other
scata3357_332	Endophyte
scata3357_272	Endophyte
scata3357_233	Other
scata3357_231	Mycorrhizal
scata3357_230	Other
scata3357_276	Mycorrhizal
scata3357_274	Mycorrhizal
scata3357_295	Mycorrhizal
scata3357_298	Endophyte
scata3357_333	Endophyte
scata3357_281	Mycorrhizal
scata3357_244	Mycorrhizal
scata3357_219	Other
scata3357_275	Endophyte
scata3357_283	No hit
scata3357_292	Mycorrhizal
scata3357_249	Mycorrhizal
scata3357_327	Mycorrhizal
scata3357_268	Endophyte
scata3357_296	Other
scata3357_307	No hit
scata3357_318	No hit
scata3357_284	Endophyte
scata3357_293	Mycorrhizal
scata3357_288	Mycorrhizal
scata3357_264	Mycorrhizal
scata3357_302	Endophyte
scata3357_257	Mycorrhizal
scata3357_263	Mixed
scata3357_353	Endophyte
scata3357_282	Other
scata3357_279	Mycorrhizal
scata3357_300	Endophyte
scata3357_313	No hit
scata3357_285	Mycorrhizal
scata3357_337	Mixed
scata3357_310	Other
scata3357_265	Mycorrhizal
scata3357_267	Mycorrhizal
scata3357_280	Endophyte
scata3357_357	Endophyte
scata3357_306	Endophyte
scata3357_290	Mycorrhizal
scata3357_304	Mycorrhizal
scata3357_343	Endophyte
scata3357_335	Other

scata3357_348	Mycorrhizal
scata3357_299	Mycorrhizal
scata3357_349	Endophyte
scata3357_297	Mycorrhizal
scata3357_309	Mycorrhizal
scata3357_311	No hit
scata3357_328	No hit
scata3357_320	Mycorrhizal
scata3357_378	Endophyte
scata3357_321	Mycorrhizal
scata3357_325	Endophyte
scata3357_372	Mixed
scata3357_301	Endophyte
scata3357_303	Mycorrhizal
scata3357_323	Mycorrhizal
scata3357_324	Mycorrhizal
scata3357_330	Mycorrhizal
scata3357_270	Other
scata3357_308	Mycorrhizal
scata3357_316	Mycorrhizal
scata3357_334	Mycorrhizal
scata3357_340	Endophyte
scata3357_344	Endophyte
scata3357_341	Endophyte
scata3357_319	Mycorrhizal
scata3357_359	Endophyte
scata3357_317	Mycorrhizal
scata3357_315	Mycorrhizal
scata3357_314	Mycorrhizal
scata3357_355	No hit
scata3357_312	Endophyte
scata3357_326	Endophyte
scata3357_345	Mycorrhizal
scata3357_347	No hit
scata3357_305	Mycorrhizal
scata3357_354	No hit
scata3357_360	Endophyte
scata3357_356	Endophyte
scata3357_433	Other
scata3357_369	No hit
scata3357_486	No hit
scata3357_351	Other
scata3357_350	Mycorrhizal
scata3357_384	Endophyte
scata3357_358	Mycorrhizal
scata3357_336	Mycorrhizal
scata3357_400	Endophyte
scata3357_329	Mycorrhizal
scata3357_386	No hit
scata3357_338	Mycorrhizal
scata3357_374	No hit
scata3357_404	Endophyte
scata3357_342	Mycorrhizal

scata3357_346	Mycorrhizal
scata3357_361	Mycorrhizal
scata3357_373	Other
scata3357_352	Mycorrhizal
scata3357_368	Mycorrhizal
scata3357_417	No hit
scata3357_446	No hit
scata3357_339	Mycorrhizal
scata3357_398	Mycorrhizal
scata3357_399	Endophyte
scata3357_406	Mycorrhizal
scata3357_397	No hit
scata3357_419	No hit
scata3357_371	Endophyte
scata3357_465	No hit
scata3357_468	Endophyte
scata3357_362	Mycorrhizal
scata3357_388	Mycorrhizal
scata3357_383	Endophyte
scata3357_382	Mycorrhizal
scata3357_389	Endophyte
scata3357_391	Other
scata3357_413	Mycorrhizal
scata3357_438	Endophyte
scata3357_473	Endophyte
scata3357_375	Mycorrhizal
scata3357_390	Endophyte
scata3357_416	No hit
scata3357_464	No hit
scata3357_385	Mycorrhizal
scata3357_440	Endophyte
scata3357_379	Mycorrhizal
scata3357_411	No hit
scata3357_441	Mycorrhizal
scata3357_393	Other
scata3357_370	Mycorrhizal
scata3357_415	Other
scata3357_376	Mycorrhizal
scata3357_414	Mycorrhizal
scata3357_364	Mycorrhizal
scata3357_428	Other
scata3357_536	No hit
scata3357_366	Mycorrhizal
scata3357_439	No hit
scata3357_430	Endophyte
scata3357_450	No hit
scata3357_480	Endophyte
scata3357_403	Endophyte
scata3357_460	Endophyte
scata3357_485	No hit
scata3357_488	Endophyte
scata3357_387	Endophyte
scata3357_407	Mycorrhizal

scata3357_461	Mycorrhizal
scata3357_381	Mycorrhizal
scata3357_396	No hit
scata3357_475	Endophyte
scata3357_402	Endophyte
scata3357_491	Mycorrhizal
scata3357_493	Other
scata3357_505	No hit
scata3357_392	Mycorrhizal
scata3357_449	No hit
scata3357_466	Mixed
scata3357_395	Mycorrhizal
scata3357_435	Mycorrhizal
scata3357_483	Other
scata3357_484	Endophyte
scata3357_487	Mycorrhizal
scata3357_377	No hit
scata3357_418	Other
scata3357_434	Mycorrhizal
scata3357_442	Mycorrhizal
scata3357_457	Endophyte
scata3357_409	Mycorrhizal
scata3357_427	No hit
scata3357_452	Mycorrhizal
scata3357_405	No hit
scata3357_429	Mycorrhizal
scata3357_448	No hit
scata3357_410	Other
scata3357_421	Mycorrhizal
scata3357_436	Other
scata3357_437	Mycorrhizal
scata3357_444	Endophyte
scata3357_513	Endophyte
scata3357_423	Mycorrhizal
scata3357_426	Mycorrhizal
scata3357_425	Endophyte
scata3357_518	Mycorrhizal
scata3357_380	Mycorrhizal
scata3357_422	Mycorrhizal
scata3357_445	Mycorrhizal
scata3357_476	No hit
scata3357_478	No hit
scata3357_424	Mycorrhizal
scata3357_523	No hit
scata3357_506	Other
scata3357_520	Endophyte
scata3357_472	Mycorrhizal
scata3357_510	No hit
scata3357_524	Other
scata3357_458	Mycorrhizal
scata3357_467	Mixed
scata3357_477	Mycorrhizal
scata3357_479	Mycorrhizal

scata3357_595	Endophyte
scata3357_408	Mycorrhizal
scata3357_420	Other
scata3357_470	Other
scata3357_492	No hit
scata3357_556	No hit
scata3357_562	No hit
scata3357_453	Mycorrhizal
scata3357_459	Mycorrhizal
scata3357_498	Other
scata3357_508	Mycorrhizal
scata3357_519	Endophyte
scata3357_456	Mycorrhizal
scata3357_499	Mycorrhizal
scata3357_471	Endophyte
scata3357_495	Mycorrhizal
scata3357_496	Endophyte
scata3357_501	Endophyte
scata3357_528	No hit
scata3357_482	Mycorrhizal
scata3357_527	Endophyte
scata3357_561	Other
scata3357_481	Endophyte
scata3357_522	No hit
scata3357_543	Endophyte
scata3357_447	No hit
scata3357_511	No hit
scata3357_537	No hit
scata3357_469	No hit
scata3357_431	Other
scata3357_568	Endophyte
scata3357_659	No hit
scata3357_503	Endophyte
scata3357_549	No hit
scata3357_694	No hit
scata3357_590	No hit
scata3357_609	No hit
scata3357_474	Other
scata3357_512	Mycorrhizal
scata3357_555	No hit
scata3357_576	Mixed
scata3357_500	No hit
scata3357_509	Mycorrhizal
scata3357_664	No hit
scata3357_573	Other
scata3357_601	Mycorrhizal
scata3357_515	Mycorrhizal
scata3357_517	No hit
scata3357_605	Mycorrhizal
scata3357_443	Mycorrhizal
scata3357_497	Mycorrhizal
scata3357_529	Other
scata3357_541	Mycorrhizal

scata3357_602	Endophyte
scata3357_454	Mycorrhizal
scata3357_507	Mycorrhizal
scata3357_531	Endophyte
scata3357_571	Mixed
scata3357_451	Mycorrhizal
scata3357_494	Mycorrhizal
scata3357_544	Mycorrhizal
scata3357_579	Other
scata3357_463	No hit
scata3357_504	No hit
scata3357_539	No hit
scata3357_551	No hit
scata3357_586	Endophyte
scata3357_612	Endophyte
scata3357_735	No hit
scata3357_489	Mycorrhizal
scata3357_554	No hit
scata3357_583	Mycorrhizal
scata3357_542	Other
scata3357_545	Endophyte
scata3357_548	Mycorrhizal
scata3357_584	Mycorrhizal
scata3357_697	Endophyte
scata3357_490	Mycorrhizal
scata3357_526	Other
scata3357_550	Mycorrhizal
scata3357_681	No hit
scata3357_538	Other
scata3357_567	No hit
scata3357_607	Other
scata3357_560	Endophyte
scata3357_600	No hit
scata3357_547	Other
scata3357_592	Endophyte
scata3357_617	Endophyte
scata3357_514	Mycorrhizal
scata3357_525	Mycorrhizal
scata3357_530	Mycorrhizal
scata3357_557	Mycorrhizal
scata3357_574	Mycorrhizal
scata3357_588	Endophyte
scata3357_604	Mycorrhizal
scata3357_533	Mycorrhizal
scata3357_587	Mycorrhizal
scata3357_596	Other
scata3357_606	Other
scata3357_614	No hit
scata3357_559	No hit
scata3357_650	Other
scata3357_656	Mixed
scata3357_585	No hit
scata3357_611	Other

scata3357_652	Endophyte
scata3357_535	Other
scata3357_563	Mycorrhizal
scata3357_580	Other
scata3357_564	Mycorrhizal
scata3357_566	Mycorrhizal
scata3357_575	No hit
scata3357_593	Endophyte
scata3357_673	Other
scata3357_691	Endophyte
scata3357_572	No hit
scata3357_589	No hit
scata3357_594	Endophyte
scata3357_619	Other
scata3357_629	Endophyte
scata3357_711	Endophyte
scata3357_798	Mycorrhizal
scata3357_570	Mycorrhizal
scata3357_577	No hit
scata3357_635	Endophyte
scata3357_658	Endophyte
scata3357_552	Mycorrhizal
scata3357_618	Mycorrhizal
scata3357_634	Endophyte
scata3357_638	Endophyte
scata3357_640	No hit
scata3357_689	Endophyte
scata3357_603	No hit
scata3357_624	No hit
scata3357_663	Endophyte
scata3357_872	No hit
scata3357_521	Other
scata3357_569	Mycorrhizal
scata3357_641	No hit
scata3357_698	Endophyte
scata3357_738	Other
scata3357_654	Mycorrhizal
scata3357_674	Other
scata3357_685	Mixed
scata3357_701	No hit
scata3357_718	Endophyte
scata3357_546	Mycorrhizal
scata3357_598	Endophyte
scata3357_677	Mycorrhizal
scata3357_803	Endophyte
scata3357_613	Mycorrhizal
scata3357_643	No hit
scata3357_680	No hit
scata3357_687	Endophyte
scata3357_743	No hit
scata3357_558	Mycorrhizal
scata3357_623	Mycorrhizal
scata3357_646	Endophyte

scata3357_714	Endophyte
scata3357_787	Endophyte
scata3357_814	Endophyte
scata3357_565	Other
scata3357_636	No hit
scata3357_655	Endophyte
scata3357_672	Endophyte
scata3357_682	Mycorrhizal
scata3357_730	Endophyte
scata3357_639	No hit
scata3357_723	Other
scata3357_731	Mycorrhizal
scata3357_733	Mixed
scata3357_553	No hit
scata3357_622	Mycorrhizal
scata3357_637	Mycorrhizal
scata3357_645	Endophyte
scata3357_657	Mycorrhizal
scata3357_737	Endophyte
scata3357_741	Mycorrhizal
scata3357_813	Endophyte
scata3357_581	No hit
scata3357_626	No hit
scata3357_632	No hit
scata3357_651	Other
scata3357_662	Endophyte
scata3357_751	Endophyte
scata3357_756	No hit
scata3357_817	No hit
scata3357_597	Mycorrhizal
scata3357_660	No hit
scata3357_661	Mycorrhizal
scata3357_665	No hit
scata3357_699	Mycorrhizal
scata3357_727	Mixed
scata3357_744	Endophyte
scata3357_865	No hit
scata3357_630	Endophyte
scata3357_642	Other
scata3357_671	Mycorrhizal
scata3357_690	Endophyte
scata3357_702	Endophyte
scata3357_736	No hit
scata3357_752	No hit
scata3357_778	Other
scata3357_582	Other
scata3357_610	Other
scata3357_615	Endophyte
scata3357_625	Mycorrhizal
scata3357_649	Other
scata3357_679	Mycorrhizal
scata3357_688	No hit
scata3357_713	Mixed

scata3357_760	Mycorrhizal
scata3357_793	Other
scata3357_800	No hit
scata3357_812	No hit
scata3357_628	Mycorrhizal
scata3357_644	Mycorrhizal
scata3357_684	Endophyte
scata3357_692	No hit
scata3357_721	Other
scata3357_740	Other
scata3357_791	Endophyte
scata3357_653	No hit
scata3357_667	Other
scata3357_719	No hit
scata3357_788	No hit
scata3357_789	Other
scata3357_866	Endophyte
scata3357_902	No hit
scata3357_647	Other
scata3357_678	Other
scata3357_686	Mycorrhizal
scata3357_693	No hit
scata3357_749	Other
scata3357_750	Mycorrhizal
scata3357_753	No hit
scata3357_840	Other
scata3357_861	No hit
scata3357_934	Endophyte
scata3357_666	Endophyte
scata3357_700	Endophyte
scata3357_710	Endophyte
scata3357_725	Other
scata3357_726	Mycorrhizal
scata3357_728	No hit
scata3357_729	Other
scata3357_775	Endophyte
scata3357_801	Mixed
scata3357_886	No hit
scata3357_631	No hit
scata3357_633	Other
scata3357_683	No hit
scata3357_724	Other
scata3357_734	Endophyte
scata3357_759	Endophyte
scata3357_762	No hit
scata3357_790	No hit
scata3357_815	Mycorrhizal
scata3357_838	No hit
scata3357_842	Endophyte
scata3357_871	Endophyte
scata3357_887	No hit
scata3357_712	Mycorrhizal
scata3357_715	No hit

scata3357_716	No hit
scata3357_720	Mycorrhizal
scata3357_764	Mycorrhizal
scata3357_766	Other
scata3357_837	Endophyte
scata3357_859	Mycorrhizal
scata3357_862	Other
scata3357_915	Endophyte
scata3357_1050	No hit
scata3357_648	Mycorrhizal
scata3357_669	Mycorrhizal
scata3357_746	Endophyte
scata3357_768	Other
scata3357_868	Endophyte
scata3357_675	Other
scata3357_745	No hit
scata3357_844	Endophyte
scata3357_851	Other
scata3357_922	No hit
scata3357_707	No hit
scata3357_717	Other
scata3357_754	No hit
scata3357_772	Mixed
scata3357_774	Other
scata3357_779	Mycorrhizal
scata3357_807	No hit
scata3357_829	Other
scata3357_832	Mycorrhizal
scata3357_843	Endophyte
scata3357_897	Mixed
scata3357_916	Endophyte
scata3357_1026	No hit
scata3357_695	Mycorrhizal
scata3357_705	Mycorrhizal
scata3357_708	Mycorrhizal
scata3357_732	Endophyte
scata3357_742	Mycorrhizal
scata3357_748	No hit
scata3357_822	No hit
scata3357_839	Endophyte
scata3357_854	No hit
scata3357_1096	No hit
scata3357_668	Mycorrhizal
scata3357_676	No hit
scata3357_758	Other
scata3357_763	Other
scata3357_780	No hit
scata3357_797	No hit
scata3357_849	No hit
scata3357_881	Other
scata3357_951	Endophyte
scata3357_957	No hit
scata3357_1108	Endophyte

scata3357_755	Mixed
scata3357_769	Other
scata3357_770	Other
scata3357_796	Mycorrhizal
scata3357_810	Other
scata3357_811	Other
scata3357_858	Mycorrhizal
scata3357_879	No hit
scata3357_891	Endophyte
scata3357_892	No hit
scata3357_906	Mycorrhizal
scata3357_939	Endophyte
scata3357_942	No hit
scata3357_1188	Mixed
scata3357_773	Other
scata3357_864	Mycorrhizal
scata3357_878	Endophyte
scata3357_882	No hit
scata3357_910	Other
scata3357_921	No hit
scata3357_1074	Endophyte
scata3357_1098	Other
scata3357_825	No hit
scata3357_830	Mycorrhizal
scata3357_833	No hit
scata3357_841	Endophyte
scata3357_877	No hit
scata3357_894	No hit
scata3357_900	Mycorrhizal
scata3357_919	Endophyte
scata3357_959	Mixed
scata3357_1008	No hit
scata3357_1027	No hit
scata3357_1066	No hit
scata3357_703	No hit
scata3357_771	No hit
scata3357_792	Endophyte
scata3357_805	Mixed
scata3357_821	Endophyte
scata3357_827	Other
scata3357_857	Other
scata3357_867	Mycorrhizal
scata3357_874	Endophyte
scata3357_896	Other
scata3357_945	Endophyte
scata3357_999	No hit
scata3357_1045	No hit
scata3357_709	Mixed
scata3357_799	Endophyte
scata3357_808	Mycorrhizal
scata3357_818	No hit
scata3357_828	Mixed
scata3357_863	No hit

scata3357_890	No hit
scata3357_931	No hit
scata3357_944	No hit
scata3357_947	No hit
scata3357_949	No hit
scata3357_958	Mycorrhizal
scata3357_968	Endophyte
scata3357_971	Endophyte
scata3357_1002	Endophyte
scata3357_1009	No hit
scata3357_1010	Other
scata3357_777	Mycorrhizal
scata3357_781	No hit
scata3357_785	Mycorrhizal
scata3357_848	Mycorrhizal
scata3357_918	No hit
scata3357_932	No hit
scata3357_943	No hit
scata3357_964	No hit
scata3357_982	Other
scata3357_1012	Mycorrhizal
scata3357_1047	Endophyte
scata3357_1057	Endophyte
scata3357_1071	No hit
scata3357_1212	Other
scata3357_704	Mycorrhizal
scata3357_776	Mycorrhizal
scata3357_850	Other
scata3357_856	Mycorrhizal
scata3357_870	No hit
scata3357_893	Endophyte
scata3357_905	No hit
scata3357_938	Mycorrhizal
scata3357_950	No hit
scata3357_963	Endophyte
scata3357_973	Other
scata3357_1024	Endophyte
scata3357_1035	No hit
scata3357_1067	Mycorrhizal
scata3357_1124	Endophyte
scata3357_1133	Mixed
scata3357_809	Endophyte
scata3357_819	Endophyte
scata3357_836	Mycorrhizal
scata3357_846	No hit
scata3357_852	Mycorrhizal
scata3357_853	Endophyte
scata3357_920	No hit
scata3357_965	Endophyte
scata3357_972	Endophyte
scata3357_1173	Mixed
scata3357_1224	Other
scata3357_761	Mycorrhizal

scata3357_794	Endophyte
scata3357_816	Endophyte
scata3357_820	Mixed
scata3357_824	No hit
scata3357_888	No hit
scata3357_895	No hit
scata3357_901	Endophyte
scata3357_936	No hit
scata3357_979	Endophyte
scata3357_995	Other
scata3357_1001	Endophyte
scata3357_1020	Other
scata3357_1023	No hit
scata3357_1077	Other
scata3357_1086	No hit
scata3357_1110	No hit
scata3357_1144	No hit
scata3357_1177	Other
scata3357_1227	Mixed
scata3357_823	No hit
scata3357_826	No hit
scata3357_873	No hit
scata3357_909	Other
scata3357_912	Other
scata3357_941	No hit
scata3357_946	Other
scata3357_961	No hit
scata3357_966	Other
scata3357_970	Other
scata3357_980	No hit
scata3357_985	Mycorrhizal
scata3357_998	No hit
scata3357_1005	Mixed
scata3357_1015	Mycorrhizal
scata3357_1030	Endophyte
scata3357_1043	Mycorrhizal
scata3357_1051	Mycorrhizal
scata3357_1058	Mixed
scata3357_1062	No hit
scata3357_1063	Endophyte
scata3357_1078	Endophyte
scata3357_1154	No hit
scata3357_1209	Endophyte
scata3357_1236	Mycorrhizal
scata3357_1310	No hit
scata3357_1340	No hit
scata3357_1354	Other
scata3357_795	No hit
scata3357_876	Other
scata3357_885	No hit
scata3357_903	Mycorrhizal
scata3357_911	Mycorrhizal
scata3357_925	No hit

scata3357_929	Other
scata3357_955	No hit
scata3357_956	No hit
scata3357_960	Mycorrhizal
scata3357_977	Endophyte
scata3357_983	Other
scata3357_987	Endophyte
scata3357_1000	Mixed
scata3357_1006	Other
scata3357_1014	Endophyte
scata3357_1028	No hit
scata3357_1031	No hit
scata3357_1056	Endophyte
scata3357_1060	Mycorrhizal
scata3357_1068	Endophyte
scata3357_1075	Other
scata3357_1088	No hit
scata3357_1092	No hit
scata3357_1117	No hit
scata3357_1128	Mixed
scata3357_1135	Mycorrhizal
scata3357_1138	Other
scata3357_1241	No hit
scata3357_1300	Mycorrhizal
scata3357_1542	Endophyte
scata3357_847	No hit
scata3357_869	No hit
scata3357_898	No hit
scata3357_914	Endophyte
scata3357_933	No hit
scata3357_935	Other
scata3357_975	Endophyte
scata3357_976	Other
scata3357_981	Mycorrhizal
scata3357_1017	Other
scata3357_1054	No hit
scata3357_1081	No hit
scata3357_1132	Mycorrhizal
scata3357_1178	No hit
scata3357_1223	No hit
scata3357_1244	No hit
scata3357_1260	No hit
scata3357_831	Mycorrhizal
scata3357_880	No hit
scata3357_924	Mycorrhizal
scata3357_926	Mycorrhizal
scata3357_930	Mycorrhizal
scata3357_974	Other
scata3357_988	Mycorrhizal
scata3357_1042	Mycorrhizal
scata3357_1082	Mycorrhizal
scata3357_1084	No hit
scata3357_1101	Other

scata3357_1102	No hit
scata3357_1119	Mycorrhizal
scata3357_1122	No hit
scata3357_1141	Mycorrhizal
scata3357_1157	No hit
scata3357_1271	No hit
scata3357_1295	Mycorrhizal
scata3357_1458	No hit
scata3357_1716	Other
scata3357_904	No hit
scata3357_927	Mycorrhizal
scata3357_928	Endophyte
scata3357_940	Mycorrhizal
scata3357_990	Other
scata3357_994	Mixed
scata3357_997	No hit
scata3357_1036	No hit
scata3357_1041	Mycorrhizal
scata3357_1089	Mycorrhizal
scata3357_1090	No hit
scata3357_1147	No hit
scata3357_1161	No hit
scata3357_1184	Other
scata3357_1192	No hit
scata3357_1203	Other
scata3357_1226	No hit
scata3357_1314	Mycorrhizal
scata3357_1809	Mycorrhizal
scata3357_907	Mycorrhizal
scata3357_917	No hit
scata3357_962	Mycorrhizal
scata3357_986	Other
scata3357_991	Endophyte
scata3357_1018	No hit
scata3357_1019	No hit
scata3357_1037	Endophyte
scata3357_1040	Other
scata3357_1052	Other
scata3357_1055	Mycorrhizal
scata3357_1069	Mycorrhizal
scata3357_1073	No hit
scata3357_1093	Endophyte
scata3357_1120	Mixed
scata3357_1130	Other
scata3357_1149	Mycorrhizal
scata3357_1158	No hit
scata3357_1160	Other
scata3357_1163	Other
scata3357_1165	Mixed
scata3357_1172	No hit
scata3357_1176	Other
scata3357_1181	Mycorrhizal
scata3357_1201	Mycorrhizal

scata3357_1207	No hit
scata3357_1219	Endophyte
scata3357_1252	Mixed
scata3357_1276	Mixed
scata3357_1437	No hit
scata3357_1484	No hit
scata3357_1518	Endophyte
scata3357_1521	Endophyte
scata3357_1534	No hit
scata3357_1838	Mycorrhizal
scata3357_989	Mycorrhizal
scata3357_993	Mycorrhizal
scata3357_1033	No hit
scata3357_1038	No hit
scata3357_1044	Endophyte
scata3357_1049	Other
scata3357_1070	No hit
scata3357_1076	No hit
scata3357_1083	Mycorrhizal
scata3357_1095	Endophyte
scata3357_1106	No hit
scata3357_1112	Mixed
scata3357_1113	Mycorrhizal
scata3357_1123	Mycorrhizal
scata3357_1126	Endophyte
scata3357_1131	No hit
scata3357_1139	Other
scata3357_1143	Endophyte
scata3357_1151	Other
scata3357_1183	No hit
scata3357_1191	No hit
scata3357_1198	No hit
scata3357_1204	No hit
scata3357_1215	Other
scata3357_1216	Mycorrhizal
scata3357_1225	Mycorrhizal
scata3357_1231	No hit
scata3357_1235	Mycorrhizal
scata3357_1239	No hit
scata3357_1240	No hit
scata3357_1256	Endophyte
scata3357_1279	No hit
scata3357_1351	No hit
scata3357_1375	Mycorrhizal
scata3357_1385	Endophyte
scata3357_1401	Mycorrhizal
scata3357_1426	Mycorrhizal
scata3357_1441	No hit
scata3357_1445	No hit
scata3357_1498	Mixed
scata3357_1506	No hit
scata3357_1524	Mycorrhizal
scata3357_1535	Other

scata3357_2276	Endophyte
scata3357_2777	Other
scata3357_954	Mycorrhizal
scata3357_1007	Other
scata3357_1013	Mycorrhizal
scata3357_1025	Other
scata3357_1029	Mycorrhizal
scata3357_1048	No hit
scata3357_1053	Other
scata3357_1059	Endophyte
scata3357_1065	Mycorrhizal
scata3357_1072	No hit
scata3357_1079	No hit
scata3357_1080	Endophyte
scata3357_1085	Mycorrhizal
scata3357_1091	Mycorrhizal
scata3357_1097	No hit
scata3357_1107	Endophyte
scata3357_1115	No hit
scata3357_1137	Mycorrhizal
scata3357_1170	No hit
scata3357_1182	Mycorrhizal
scata3357_1187	Endophyte
scata3357_1190	Other
scata3357_1199	Other
scata3357_1206	Other
scata3357_1213	Mixed
scata3357_1233	Endophyte
scata3357_1254	Other
scata3357_1263	Mycorrhizal
scata3357_1264	Endophyte
scata3357_1275	No hit
scata3357_1277	No hit
scata3357_1280	Mycorrhizal
scata3357_1281	Mixed
scata3357_1304	Mycorrhizal
scata3357_1315	No hit
scata3357_1349	Mycorrhizal
scata3357_1368	Mixed
scata3357_1371	Endophyte
scata3357_1396	Endophyte
scata3357_1424	Endophyte
scata3357_1485	Mycorrhizal
scata3357_1488	Other
scata3357_1692	No hit
scata3357_1926	Other
scata3357_1960	Endophyte
scata3357_1032	Other
scata3357_1046	Other
scata3357_1064	Endophyte
scata3357_1087	Mycorrhizal
scata3357_1114	Mycorrhizal
scata3357_1142	Mycorrhizal

scata3357_1152	Endophyte
scata3357_1168	Other
scata3357_1171	Mycorrhizal
scata3357_1186	Endophyte
scata3357_1211	Endophyte
scata3357_1245	Endophyte
scata3357_1246	Mixed
scata3357_1250	No hit
scata3357_1266	No hit
scata3357_1288	No hit
scata3357_1311	No hit
scata3357_1316	No hit
scata3357_1317	No hit
scata3357_1319	Other
scata3357_1331	No hit
scata3357_1332	No hit
scata3357_1342	No hit
scata3357_1344	No hit
scata3357_1348	No hit
scata3357_1353	No hit
scata3357_1382	Mycorrhizal
scata3357_1389	Endophyte
scata3357_1390	Endophyte
scata3357_1395	Endophyte
scata3357_1414	Mycorrhizal
scata3357_1469	Endophyte
scata3357_1477	Endophyte
scata3357_1482	Other
scata3357_1494	Other
scata3357_1536	No hit
scata3357_1558	Endophyte
scata3357_1612	No hit
scata3357_1711	Endophyte
scata3357_1789	Mycorrhizal
scata3357_1792	Other
scata3357_1812	No hit
scata3357_1844	Endophyte
scata3357_2286	Other
scata3357_1100	Endophyte
scata3357_1129	Mycorrhizal
scata3357_1148	No hit
scata3357_1156	Mycorrhizal
scata3357_1166	Mycorrhizal
scata3357_1169	No hit
scata3357_1175	Other
scata3357_1180	No hit
scata3357_1189	No hit
scata3357_1196	No hit
scata3357_1197	Mycorrhizal
scata3357_1205	No hit
scata3357_1214	Endophyte
scata3357_1217	Endophyte
scata3357_1221	No hit

scata3357_1243	No hit
scata3357_1257	No hit
scata3357_1285	No hit
scata3357_1294	No hit
scata3357_1298	Mycorrhizal
scata3357_1299	Mycorrhizal
scata3357_1309	Endophyte
scata3357_1312	Mycorrhizal
scata3357_1318	No hit
scata3357_1334	No hit
scata3357_1335	Mixed
scata3357_1339	Endophyte
scata3357_1341	Other
scata3357_1347	No hit
scata3357_1358	Mixed
scata3357_1384	Other
scata3357_1393	Other
scata3357_1447	Other
scata3357_1459	Endophyte
scata3357_1465	No hit
scata3357_1473	No hit
scata3357_1564	Endophyte
scata3357_1577	No hit
scata3357_1588	Other
scata3357_1602	No hit
scata3357_1727	Endophyte
scata3357_1771	No hit
scata3357_1928	Endophyte
scata3357_2092	Endophyte
scata3357_2335	Endophyte
scata3357_2714	No hit
scata3357_11796	Endophyte
scata3357_1105	Mycorrhizal
scata3357_1155	No hit
scata3357_1162	Other
scata3357_1193	No hit
scata3357_1195	Mycorrhizal
scata3357_1208	No hit
scata3357_1218	Endophyte
scata3357_1220	No hit
scata3357_1222	No hit
scata3357_1234	Mycorrhizal
scata3357_1255	No hit
scata3357_1284	Other
scata3357_1286	Endophyte
scata3357_1292	Endophyte
scata3357_1293	No hit
scata3357_1302	No hit
scata3357_1305	Mycorrhizal
scata3357_1308	Mycorrhizal
scata3357_1320	Other
scata3357_1322	Mycorrhizal
scata3357_1323	No hit

scata3357_1325	Endophyte
scata3357_1330	Other
scata3357_1343	Mycorrhizal
scata3357_1350	No hit
scata3357_1361	Mycorrhizal
scata3357_1365	No hit
scata3357_1370	Other
scata3357_1372	Endophyte
scata3357_1373	Mixed
scata3357_1374	Endophyte
scata3357_1377	Endophyte
scata3357_1402	No hit
scata3357_1409	Endophyte
scata3357_1418	Endophyte
scata3357_1425	Other
scata3357_1434	Mycorrhizal
scata3357_1461	Endophyte
scata3357_1478	No hit
scata3357_1480	Mycorrhizal
scata3357_1486	No hit
scata3357_1487	Mycorrhizal
scata3357_1501	No hit
scata3357_1504	No hit
scata3357_1510	Other
scata3357_1528	No hit
scata3357_1530	No hit
scata3357_1566	Mycorrhizal
scata3357_1569	Mycorrhizal
scata3357_1585	Mixed
scata3357_1599	No hit
scata3357_1627	No hit
scata3357_1632	Other
scata3357_1634	Endophyte
scata3357_1646	Endophyte
scata3357_1647	Other
scata3357_1662	Endophyte
scata3357_1670	Endophyte
scata3357_1673	Endophyte
scata3357_1706	Endophyte
scata3357_1714	No hit
scata3357_1733	Endophyte
scata3357_1748	Other
scata3357_1758	Endophyte
scata3357_1818	Endophyte
scata3357_1829	No hit
scata3357_1831	Endophyte
scata3357_1860	Endophyte
scata3357_1935	Mycorrhizal
scata3357_2043	No hit
scata3357_2149	No hit
scata3357_2160	No hit
scata3357_2165	No hit
scata3357_2183	Endophyte

scata3357_2358	No hit
scata3357_2597	No hit
scata3357_6439	No hit
scata3357_1237	No hit
scata3357_1258	No hit
scata3357_1262	No hit
scata3357_1267	No hit
scata3357_1268	No hit
scata3357_1270	No hit
scata3357_1273	Mycorrhizal
scata3357_1278	No hit
scata3357_1291	Other
scata3357_1296	Endophyte
scata3357_1297	Mycorrhizal
scata3357_1301	Other
scata3357_1303	Mycorrhizal
scata3357_1306	Other
scata3357_1307	Mycorrhizal
scata3357_1326	Mixed
scata3357_1336	Other
scata3357_1337	No hit
scata3357_1346	Endophyte
scata3357_1355	No hit
scata3357_1359	Mycorrhizal
scata3357_1367	Other
scata3357_1379	No hit
scata3357_1383	Other
scata3357_1388	Other
scata3357_1400	No hit
scata3357_1406	Other
scata3357_1411	No hit
scata3357_1412	No hit
scata3357_1415	No hit
scata3357_1421	No hit
scata3357_1431	Other
scata3357_1432	Mixed
scata3357_1438	No hit
scata3357_1439	Endophyte
scata3357_1448	Mixed
scata3357_1460	No hit
scata3357_1463	Mycorrhizal
scata3357_1467	No hit
scata3357_1479	Endophyte
scata3357_1481	No hit
scata3357_1489	Endophyte
scata3357_1492	No hit
scata3357_1495	Other
scata3357_1497	Endophyte
scata3357_1499	No hit
scata3357_1511	No hit
scata3357_1512	No hit
scata3357_1513	Mycorrhizal
scata3357_1514	No hit

scata3357_1515	No hit
scata3357_1517	No hit
scata3357_1519	No hit
scata3357_1523	Other
scata3357_1525	Other
scata3357_1540	Endophyte
scata3357_1551	No hit
scata3357_1552	No hit
scata3357_1563	No hit
scata3357_1572	Other
scata3357_1578	Endophyte
scata3357_1598	No hit
scata3357_1603	No hit
scata3357_1608	Endophyte
scata3357_1629	Other
scata3357_1637	No hit
scata3357_1650	Other
scata3357_1674	No hit
scata3357_1689	No hit
scata3357_1691	No hit
scata3357_1713	Other
scata3357_1721	Other
scata3357_1724	No hit
scata3357_1728	No hit
scata3357_1736	Other
scata3357_1753	Mycorrhizal
scata3357_1764	No hit
scata3357_1769	No hit
scata3357_1779	Endophyte
scata3357_1790	No hit
scata3357_1793	Other
scata3357_1794	No hit
scata3357_1795	No hit
scata3357_1796	Mycorrhizal
scata3357_1798	No hit
scata3357_1820	Endophyte
scata3357_1826	Endophyte
scata3357_1847	Endophyte
scata3357_1851	Endophyte
scata3357_1870	No hit
scata3357_1880	Mixed
scata3357_1933	Other
scata3357_1979	Other
scata3357_2090	No hit
scata3357_2120	Other
scata3357_2159	Other
scata3357_2175	No hit
scata3357_2317	No hit
scata3357_2361	Endophyte
scata3357_2371	No hit
scata3357_2491	No hit
scata3357_2612	Mycorrhizal
scata3357_2702	No hit

scata3357_2709	No hit
scata3357_2756	Mixed
scata3357_3340	Mixed
scata3357_11073	Mixed
scata3357_1362	Endophyte
scata3357_1376	No hit
scata3357_1381	Mycorrhizal
scata3357_1386	No hit
scata3357_1391	No hit
scata3357_1394	Mycorrhizal
scata3357_1398	Other
scata3357_1403	No hit
scata3357_1407	Endophyte
scata3357_1408	Endophyte
scata3357_1413	Mycorrhizal
scata3357_1416	No hit
scata3357_1417	No hit
scata3357_1419	No hit
scata3357_1422	No hit
scata3357_1427	Endophyte
scata3357_1433	Mycorrhizal
scata3357_1435	Mixed
scata3357_1436	Other
scata3357_1440	Mycorrhizal
scata3357_1442	No hit
scata3357_1450	Endophyte
scata3357_1455	No hit
scata3357_1457	No hit
scata3357_1462	Mycorrhizal
scata3357_1464	Other
scata3357_1471	Endophyte
scata3357_1472	No hit
scata3357_1475	Mycorrhizal
scata3357_1476	No hit
scata3357_1483	No hit
scata3357_1491	Mycorrhizal
scata3357_1496	Endophyte
scata3357_1500	Other
scata3357_1505	Mycorrhizal
scata3357_1507	No hit
scata3357_1508	Other
scata3357_1516	No hit
scata3357_1526	No hit
scata3357_1527	No hit
scata3357_1531	Mycorrhizal
scata3357_1533	No hit
scata3357_1537	No hit
scata3357_1543	No hit
scata3357_1545	Endophyte
scata3357_1548	No hit
scata3357_1571	No hit
scata3357_1573	Mycorrhizal
scata3357_1576	Mycorrhizal

scata3357_1587	Mycorrhizal
scata3357_1594	Other
scata3357_1596	Mycorrhizal
scata3357_1600	No hit
scata3357_1604	Endophyte
scata3357_1611	Other
scata3357_1613	No hit
scata3357_1619	No hit
scata3357_1621	Mycorrhizal
scata3357_1624	Other
scata3357_1625	Other
scata3357_1626	No hit
scata3357_1630	No hit
scata3357_1636	No hit
scata3357_1640	No hit
scata3357_1641	Endophyte
scata3357_1656	Mixed
scata3357_1672	Endophyte
scata3357_1684	Other
scata3357_1686	Mycorrhizal
scata3357_1688	No hit
scata3357_1710	Endophyte
scata3357_1719	Mycorrhizal
scata3357_1720	Mycorrhizal
scata3357_1745	Mycorrhizal
scata3357_1749	No hit
scata3357_1754	Other
scata3357_1756	No hit
scata3357_1757	Mycorrhizal
scata3357_1762	No hit
scata3357_1766	Endophyte
scata3357_1773	No hit
scata3357_1788	No hit
scata3357_1797	Other
scata3357_1803	Endophyte
scata3357_1807	No hit
scata3357_1816	Mixed
scata3357_1822	Other
scata3357_1825	Other
scata3357_1841	Other
scata3357_1848	Mycorrhizal
scata3357_1856	Mycorrhizal
scata3357_1857	No hit
scata3357_1867	No hit
scata3357_1868	Endophyte
scata3357_1878	No hit
scata3357_1886	No hit
scata3357_1891	Endophyte
scata3357_1909	No hit
scata3357_1912	Other
scata3357_1934	Other
scata3357_2115	No hit
scata3357_2144	No hit

scata3357_2214	Endophyte
scata3357_2226	No hit
scata3357_2253	No hit
scata3357_2268	No hit
scata3357_2285	No hit
scata3357_2348	No hit
scata3357_2381	Mycorrhizal
scata3357_2408	No hit
scata3357_2440	No hit
scata3357_2457	No hit
scata3357_2511	Endophyte
scata3357_2600	Endophyte
scata3357_2608	No hit
scata3357_2629	Mycorrhizal
scata3357_2630	Endophyte
scata3357_2647	Endophyte
scata3357_2703	Endophyte
scata3357_2753	Endophyte
scata3357_4580	No hit
scata3357_6300	No hit
scata3357_6680	Endophyte
scata3357_9004	Mixed
scata3357_9383	Endophyte
scata3357_9667	Mixed
scata3357_13393	No hit
scata3357_1539	Mycorrhizal
scata3357_1541	Mixed
scata3357_1550	No hit
scata3357_1554	Mixed
scata3357_1555	No hit
scata3357_1559	No hit
scata3357_1560	No hit
scata3357_1561	No hit
scata3357_1562	Other
scata3357_1565	No hit
scata3357_1570	Mycorrhizal
scata3357_1574	No hit
scata3357_1584	No hit
scata3357_1590	No hit
scata3357_1593	No hit
scata3357_1601	Other
scata3357_1605	Other
scata3357_1620	Mycorrhizal
scata3357_1623	No hit
scata3357_1631	No hit
scata3357_1638	No hit
scata3357_1643	No hit
scata3357_1648	Mycorrhizal
scata3357_1649	No hit
scata3357_1653	No hit
scata3357_1658	Mixed
scata3357_1659	No hit
scata3357_1664	Endophyte

scata3357_1665	Other
scata3357_1667	Endophyte
scata3357_1668	Mycorrhizal
scata3357_1671	Mycorrhizal
scata3357_1675	Other
scata3357_1678	Mycorrhizal
scata3357_1681	Mycorrhizal
scata3357_1683	No hit
scata3357_1687	No hit
scata3357_1695	Other
scata3357_1697	Mycorrhizal
scata3357_1699	No hit
scata3357_1700	Mycorrhizal
scata3357_1703	No hit
scata3357_1704	Other
scata3357_1705	No hit
scata3357_1715	Other
scata3357_1717	Endophyte
scata3357_1718	No hit
scata3357_1726	No hit
scata3357_1730	Other
scata3357_1731	Mycorrhizal
scata3357_1734	No hit
scata3357_1739	No hit
scata3357_1751	Endophyte
scata3357_1761	Mycorrhizal
scata3357_1763	Other
scata3357_1765	Endophyte
scata3357_1767	Mycorrhizal
scata3357_1772	Other
scata3357_1775	No hit
scata3357_1776	Mycorrhizal
scata3357_1777	No hit
scata3357_1778	Other
scata3357_1780	No hit
scata3357_1783	Other
scata3357_1786	Endophyte
scata3357_1791	No hit
scata3357_1799	No hit
scata3357_1801	Mycorrhizal
scata3357_1804	Other
scata3357_1805	No hit
scata3357_1806	Endophyte
scata3357_1808	Mycorrhizal
scata3357_1810	No hit
scata3357_1815	Other
scata3357_1819	No hit
scata3357_1821	Mixed
scata3357_1832	No hit
scata3357_1840	Other
scata3357_1843	Other
scata3357_1858	No hit
scata3357_1859	No hit

scata3357_1861	No hit
scata3357_1865	No hit
scata3357_1866	Mycorrhizal
scata3357_1871	No hit
scata3357_1873	Endophyte
scata3357_1875	No hit
scata3357_1879	Endophyte
scata3357_1898	No hit
scata3357_1915	No hit
scata3357_1936	Mycorrhizal
scata3357_1946	No hit
scata3357_1954	Endophyte
scata3357_1966	Endophyte
scata3357_1984	No hit
scata3357_1987	No hit
scata3357_1997	No hit
scata3357_2009	Endophyte
scata3357_2013	Endophyte
scata3357_2018	Other
scata3357_2036	No hit
scata3357_2052	Mycorrhizal
scata3357_2071	No hit
scata3357_2079	No hit
scata3357_2126	Other
scata3357_2132	Other
scata3357_2134	No hit
scata3357_2153	Endophyte
scata3357_2162	No hit
scata3357_2185	Endophyte
scata3357_2210	Other
scata3357_2232	Other
scata3357_2246	No hit
scata3357_2281	Mixed
scata3357_2324	Endophyte
scata3357_2329	No hit
scata3357_2370	Other
scata3357_2434	No hit
scata3357_2446	Mycorrhizal
scata3357_2466	No hit
scata3357_2474	Endophyte
scata3357_2488	No hit
scata3357_2499	No hit
scata3357_2506	No hit
scata3357_2512	Endophyte
scata3357_2528	Endophyte
scata3357_2545	Other
scata3357_2552	No hit
scata3357_2556	No hit
scata3357_2578	No hit
scata3357_2579	Endophyte
scata3357_2593	No hit
scata3357_2598	Endophyte
scata3357_2626	Endophyte

scata3357_2644	No hit
scata3357_2652	Endophyte
scata3357_2711	No hit
scata3357_2712	No hit
scata3357_2734	No hit
scata3357_2748	Endophyte
scata3357_2764	No hit
scata3357_3099	No hit
scata3357_3688	Mycorrhizal
scata3357_5891	No hit
scata3357_7501	No hit
scata3357_8097	Mixed
scata3357_8256	Endophyte
scata3357_8515	Mixed
scata3357_8948	No hit
scata3357_9548	No hit
scata3357_10038	Mixed
scata3357_10160	No hit
scata3357_10311	No hit
scata3357_10531	Other
scata3357_10827	Mixed
scata3357_11421	No hit
scata3357_11455	Mixed
scata3357_11658	Mixed
scata3357_12221	Other
scata3357_12865	No hit
scata3357_1544	No hit
scata3357_1633	Endophyte
scata3357_1696	Other
scata3357_1770	Endophyte
scata3357_1885	No hit
scata3357_1887	No hit
scata3357_1889	Mycorrhizal
scata3357_1896	Endophyte
scata3357_1899	Other
scata3357_1900	No hit
scata3357_1901	Other
scata3357_1906	No hit
scata3357_1907	No hit
scata3357_1910	No hit
scata3357_1913	No hit
scata3357_1916	Mycorrhizal
scata3357_1918	Endophyte
scata3357_1920	No hit
scata3357_1924	No hit
scata3357_1931	Endophyte
scata3357_1937	No hit
scata3357_1942	No hit
scata3357_1945	Endophyte
scata3357_1948	No hit
scata3357_1949	Endophyte
scata3357_1952	Endophyte
scata3357_1958	No hit

scata3357_1963	Mixed
scata3357_1964	No hit
scata3357_1970	No hit
scata3357_1971	Endophyte
scata3357_1973	No hit
scata3357_1975	Other
scata3357_1976	Mycorrhizal
scata3357_1978	Endophyte
scata3357_1980	Other
scata3357_1990	Endophyte
scata3357_1994	Endophyte
scata3357_1998	No hit
scata3357_2001	No hit
scata3357_2003	No hit
scata3357_2006	Mycorrhizal
scata3357_2007	Other
scata3357_2011	Mixed
scata3357_2014	No hit
scata3357_2019	No hit
scata3357_2020	No hit
scata3357_2023	Mycorrhizal
scata3357_2024	Other
scata3357_2032	Mycorrhizal
scata3357_2034	Other
scata3357_2038	Mixed
scata3357_2042	Endophyte
scata3357_2045	No hit
scata3357_2051	No hit
scata3357_2054	Mycorrhizal
scata3357_2055	No hit
scata3357_2056	Other
scata3357_2057	Other
scata3357_2058	Other
scata3357_2059	No hit
scata3357_2061	Mixed
scata3357_2062	No hit
scata3357_2067	Mycorrhizal
scata3357_2068	Endophyte
scata3357_2070	No hit
scata3357_2072	No hit
scata3357_2077	Endophyte
scata3357_2085	No hit
scata3357_2086	Endophyte
scata3357_2087	No hit
scata3357_2096	Mycorrhizal
scata3357_2099	No hit
scata3357_2104	Endophyte
scata3357_2110	No hit
scata3357_2112	No hit
scata3357_2114	Endophyte
scata3357_2116	Endophyte
scata3357_2118	Endophyte
scata3357_2121	No hit

scata3357_2122	No hit
scata3357_2123	Other
scata3357_2125	No hit
scata3357_2128	Other
scata3357_2130	Mixed
scata3357_2136	Mycorrhizal
scata3357_2138	No hit
scata3357_2143	Endophyte
scata3357_2145	No hit
scata3357_2152	Mycorrhizal
scata3357_2154	Endophyte
scata3357_2167	Mixed
scata3357_2170	No hit
scata3357_2171	No hit
scata3357_2174	No hit
scata3357_2191	No hit
scata3357_2192	No hit
scata3357_2193	Mycorrhizal
scata3357_2200	Mycorrhizal
scata3357_2201	Mycorrhizal
scata3357_2202	No hit
scata3357_2203	Mycorrhizal
scata3357_2204	Endophyte
scata3357_2209	Endophyte
scata3357_2212	No hit
scata3357_2213	Endophyte
scata3357_2218	No hit
scata3357_2219	Other
scata3357_2221	Endophyte
scata3357_2224	Other
scata3357_2227	Other
scata3357_2228	No hit
scata3357_2229	No hit
scata3357_2231	Other
scata3357_2233	Mycorrhizal
scata3357_2235	Other
scata3357_2248	No hit
scata3357_2250	Endophyte
scata3357_2252	Mixed
scata3357_2254	Endophyte
scata3357_2255	Endophyte
scata3357_2256	Mixed
scata3357_2258	Endophyte
scata3357_2260	No hit
scata3357_2264	No hit
scata3357_2266	Mycorrhizal
scata3357_2272	No hit
scata3357_2274	Other
scata3357_2277	No hit
scata3357_2282	No hit
scata3357_2291	Mycorrhizal
scata3357_2292	No hit
scata3357_2296	No hit

scata3357_2297	Other
scata3357_2299	Mycorrhizal
scata3357_2303	Other
scata3357_2309	Mycorrhizal
scata3357_2311	Endophyte
scata3357_2312	Other
scata3357_2313	Other
scata3357_2320	Mixed
scata3357_2321	Other
scata3357_2323	Endophyte
scata3357_2325	Other
scata3357_2328	No hit
scata3357_2331	No hit
scata3357_2332	No hit
scata3357_2333	No hit
scata3357_2334	Endophyte
scata3357_2339	Mycorrhizal
scata3357_2340	Mixed
scata3357_2342	Mycorrhizal
scata3357_2349	Other
scata3357_2354	Endophyte
scata3357_2359	Other
scata3357_2362	Other
scata3357_2366	No hit
scata3357_2367	Endophyte
scata3357_2368	No hit
scata3357_2375	No hit
scata3357_2376	Endophyte
scata3357_2379	No hit
scata3357_2384	No hit
scata3357_2389	Mycorrhizal
scata3357_2391	No hit
scata3357_2393	Other
scata3357_2395	Mycorrhizal
scata3357_2398	Mycorrhizal
scata3357_2399	Endophyte
scata3357_2401	No hit
scata3357_2406	Mycorrhizal
scata3357_2409	No hit
scata3357_2410	No hit
scata3357_2414	Mycorrhizal
scata3357_2415	Endophyte
scata3357_2417	Mycorrhizal
scata3357_2418	Mycorrhizal
scata3357_2419	No hit
scata3357_2420	No hit
scata3357_2426	Mycorrhizal
scata3357_2427	No hit
scata3357_2429	Endophyte
scata3357_2430	Other
scata3357_2432	No hit
scata3357_2433	Endophyte
scata3357_2442	No hit

scata3357_2451	No hit
scata3357_2452	No hit
scata3357_2455	Endophyte
scata3357_2456	No hit
scata3357_2460	No hit
scata3357_2462	Endophyte
scata3357_2463	Endophyte
scata3357_2465	Endophyte
scata3357_2476	Other
scata3357_2477	No hit
scata3357_2478	Other
scata3357_2480	No hit
scata3357_2482	No hit
scata3357_2484	Endophyte
scata3357_2485	No hit
scata3357_2490	No hit
scata3357_2492	No hit
scata3357_2493	No hit
scata3357_2494	Endophyte
scata3357_2502	No hit
scata3357_2503	No hit
scata3357_2510	No hit
scata3357_2513	No hit
scata3357_2515	Mycorrhizal
scata3357_2516	No hit
scata3357_2517	Other
scata3357_2518	No hit
scata3357_2520	Endophyte
scata3357_2521	Other
scata3357_2522	No hit
scata3357_2526	Mycorrhizal
scata3357_2529	No hit
scata3357_2531	No hit
scata3357_2532	Mycorrhizal
scata3357_2533	Other
scata3357_2535	No hit
scata3357_2536	No hit
scata3357_2542	Other
scata3357_2543	No hit
scata3357_2550	No hit
scata3357_2553	No hit
scata3357_2554	Other
scata3357_2557	No hit
scata3357_2562	Other
scata3357_2565	No hit
scata3357_2566	No hit
scata3357_2568	Other
scata3357_2569	No hit
scata3357_2573	No hit
scata3357_2574	No hit
scata3357_2580	Mycorrhizal
scata3357_2581	No hit
scata3357_2586	Other
scata3357_2587	Endophyte
scata3357_2588	Other
scata3357_2594	No hit
scata3357_2599	Endophyte
scata3357_2607	Endophyte
scata3357_2609	Endophyte
scata3357_2613	Mycorrhizal
scata3357_2614	Mycorrhizal
scata3357_2615	No hit
scata3357_2618	No hit
scata3357_2619	No hit
scata3357_2622	No hit
scata3357_2623	Mycorrhizal
scata3357_2627	Other
scata3357_2628	Mycorrhizal
scata3357_2631	Endophyte
scata3357_2632	No hit
scata3357_2634	Mycorrhizal
scata3357_2635	No hit
scata3357_2641	Other
scata3357_2643	Endophyte
scata3357_2649	No hit
scata3357_2651	Other
scata3357_2657	No hit
scata3357_2658	No hit
scata3357_2663	Endophyte
scata3357_2665	Endophyte
scata3357_2673	No hit
scata3357_2675	Endophyte
scata3357_2676	Other
scata3357_2677	Mixed
scata3357_2679	Other
scata3357_2680	No hit
scata3357_2681	Mixed
scata3357_2682	Other
scata3357_2686	No hit
scata3357_2688	No hit
scata3357_2690	No hit
scata3357_2691	No hit
scata3357_2692	Endophyte
scata3357_2694	Other
scata3357_2696	Endophyte
scata3357_2697	Endophyte
scata3357_2704	No hit
scata3357_2705	No hit
scata3357_2713	No hit
scata3357_2716	No hit
scata3357_2719	No hit
scata3357_2720	Other
scata3357_2724	Endophyte
scata3357_2726	No hit
scata3357_2727	No hit
scata3357_2729	No hit

scata3357_2731	No hit
scata3357_2733	Mycorrhizal
scata3357_2735	Other
scata3357_2736	Mycorrhizal
scata3357_2737	No hit
scata3357_2743	Endophyte
scata3357_2749	Other
scata3357_2754	No hit
scata3357_2755	No hit
scata3357_2758	Other
scata3357_2759	No hit
scata3357_2761	No hit
scata3357_2763	Endophyte
scata3357_2768	No hit
scata3357_2769	Endophyte
scata3357_2770	No hit
scata3357_2771	No hit
scata3357_2775	Mixed
scata3357_2776	No hit
scata3357_2780	Other
scata3357_2898	Mixed
scata3357_3019	Mycorrhizal
scata3357_3154	No hit
scata3357_3356	Mycorrhizal
scata3357_3461	No hit
scata3357_3498	Mixed
scata3357_3528	Mixed
scata3357_3716	Other
scata3357_3802	No hit
scata3357_4114	No hit
scata3357_4145	Other
scata3357_4204	No hit
scata3357_4643	No hit
scata3357_4909	Mixed
scata3357_5192	Mixed
scata3357_5447	Mixed
scata3357_5818	No hit
scata3357_5873	No hit
scata3357_5889	Mixed
scata3357_6008	No hit
scata3357_6193	No hit
scata3357_6289	Other

scata3357_6803	No hit
scata3357_6935	No hit
scata3357_6936	No hit
scata3357_7076	No hit
scata3357_7153	Mixed
scata3357_7185	Mixed
scata3357_7394	Mixed
scata3357_7451	Mixed
scata3357_7691	No hit
scata3357_8122	No hit
scata3357_8679	No hit
scata3357_8698	No hit
scata3357_8717	No hit
scata3357_8749	No hit
scata3357_8883	No hit
scata3357_9329	Mixed
scata3357_9557	Other
scata3357_9633	No hit
scata3357_9981	Mixed
scata3357_10414	Other
scata3357_10503	Mixed
scata3357_10605	No hit
scata3357_10817	Mixed
scata3357_10934	No hit
scata3357_11132	No hit
scata3357_11248	No hit
scata3357_11328	No hit
scata3357_11441	No hit
scata3357_11560	No hit
scata3357_11570	No hit
scata3357_11769	No hit
scata3357_11913	Mixed
scata3357_12039	No hit
scata3357_12212	Other
scata3357_12225	Mixed
scata3357_12329	No hit
scata3357_12704	Mixed
scata3357_12918	Mixed
scata3357_12993	No hit
scata3357_13064	No hit
scata3357_13243	Endophyte
scata3357_13260	No hit

Table S2. Followed backward variable selection procedure based on Akaike's Information Criterion (AIC). The models with the lowest AIC values have been marked with an asterisk.

**Poisson regression model for the overall RAF richness**	
Explanatory variables included as fixed effects	*AIC*
Plant species, elevation, squared elevation, plant species * elevation, pH, percentage of soil water content, depth of the active soil layer, vegetation cover, log transformed sequencing depth	3983.8
Plant species, elevation, squared elevation, plant species * elevation, pH, percentage of soil water content, depth of the active soil layer, log transformed sequencing depth	3982.1
Plant species, elevation, squared elevation, plant species * elevation, pH, percentage of soil water content, log transformed sequencing depth	3980.8
Plant species, elevation, squared elevation, plant species * elevation, percentage of soil water content, log transformed sequencing depth	3979.7
Plant species, elevation, squared elevation, percentage of soil water content, log transformed sequencing depth	4025.3
Plant species, elevation, squared elevation, plant species * elevation, log transformed sequencing depth	3979.6*
Plant species, elevation, squared elevation, log transformed sequencing de pth	4025.2
**Poisson model for mycorrhizal species**	
Explanatory variables included as fixed effects	*AIC*
Plant species, elevation, squared elevation, plant species * elevation, pH, percentage of soil water content, depth of the active soil layer, vegetation cover, log transformed sequencing depth	2847.8
Plant species, elevation, squared elevation, plant species * elevation, pH, percentage of soil water content, depth of the active soil layer, log transformed sequencing depth	2845.8
Plant species, elevation, squared elevation, plant species * elevation, percentage of soil water content, depth of the active soil layer, log transformed sequencing depth	2844.0
Plant species, elevation, squared elevation, plant species * elevation, percentage of soil water content, log transformed sequencing depth	2842.8
Plant species, elevation, squared elevation, plant species * elevation, log transformed sequencing depth	2841.5
Plant species, elevation, squared elevation, log transformed sequencing depth	2840.8*
Elevation, squared elevation, log transformed sequencing depth	2918.1
Plant species, elevation, log transformed sequencing depth	2845.2
**Poisson model for endophytic species**	
Explanatory variables included as fixed effects	*AIC*
Plant species, elevation, squared elevation, plant species * elevation, pH, percentage of soil water content, depth of the active soil layer, vegetation cover, log transformed sequencing depth	2740.4
Plant species, elevation, squared elevation, plant species * elevation, pH, percentage of soil water content, depth of the active soil layer, log transformed sequencing depth	2738.6

Plant species, elevation, squared elevation, plant species * elevation, pH, percentage of soil water content, log transformed sequencing depth	2737.2
Plant species, elevation, squared elevation, plant species * elevation, percentage of soil water content, log transformed sequencing depth	2736.3
Plant species, elevation, squared elevation, plant species * elevation, log transformed sequencing depth	2736.1*
Plant species, elevation, squared elevation, log transformed sequencing depth	2756.1
Plant species, elevation, plant species * elevation, log transformed sequencing depth	2739.5
**Poisson model for unclassified species**	
Explanatory variables included as fixed effects	*AIC*
Plant species, elevation, squared elevation, plant species * elevation, pH, percentage of soil water content, depth of the active soil layer, vegetation cover, log transformed sequencing depth	3096.4
Plant species, elevation, squared elevation, plant species * elevation, pH, percentage of soil water content, depth of the active soil layer, log transformed sequencing depth	3094.9
Plant species, elevation, squared elevation, plant species * elevation, pH, percentage of soil water content, log transformed sequencing depth	3093.4
Plant species, elevation, squared elevation, plant species * elevation, percentage of soil water content, log transformed sequencing depth	3093.8
Plant species, elevation, squared elevation, plant species * elevation, log transformed sequencing depth	3093.3*
Plant species, elevation, squared elevation, log transformed sequencing depth	3112.7

Table S3. Community-level variation partitioning for the models assuming uniform specialization along elevation. The total explanatory power of the models (calculated as AUC values for the presence-absence models and R^2 values for the abundance models) as well as the relative effects of each variable (explained portion of the total explained variance) are provided. The models have been fitted to the presence-absence and abundance conditional on presence data. Elevation includes its linear effect as well as its unimodal effect (squared term), and soil variables include soil pH, soil water content, depth of the active soil layer and vegetation cover. The random effect corresponds to the sampling location. The corresponding figure is Figure 4 in the main manuscript.

Fungal group	Data type	Total explanatory power	Plant species	Elevation	Soil variables	Sequencing depth	Random effect
All RAF	Presence-absence	0.82	0.26	0.15	0.27	0.05	0.27
	Abundance	0.25	0.31	0.14	0.28	0.15	0.12
Mycorrhizal fungi	Presence-absence	0.80	0.11	0.26	0.41	0.04	0.18
	Abundance	0.26	0.23	0.18	0.37	0.12	0.1
Endophytic fungi	Presence-absence	0.82	0.23	0.2	0.34	0.08	0.15
	Abundance	0.25	0.29	0.14	0.31	0.16	0.1
Unclassified fungi	Presence-absence	0.85	0.4	0.13	0.3	0.05	0.12
	Abundance	0.29	0.33	0.14	0.29	0.15	0.09
